# Role of Th17 Cytokines in Airway Remodeling in Asthma and Therapy Perspectives

**DOI:** 10.3389/falgy.2022.806391

**Published:** 2022-02-02

**Authors:** Victor Margelidon-Cozzolino, Anne Tsicopoulos, Cécile Chenivesse, Patricia de Nadai

**Affiliations:** ^1^Univ. Lille, CNRS, INSERM, CHU de Lille, Institut Pasteur de Lille, Unité INSERM U1019-UMR9017-CIIL-Centre d'Infection et d'Immunité de Lille, Lille, France; ^2^CRISALIS (Clinical Research Initiative in Severe Asthma: a Lever for Innovation & Science), F-CRIN Network, INSERM US015, Toulouse, France

**Keywords:** asthma, airway remodeling, Th17 inflammation, interleukine-17, interleukine-22

## Abstract

Airway remodeling is a frequent pathological feature of severe asthma leading to permanent airway obstruction in up to 50% of cases and to respiratory disability. Although structural changes related to airway remodeling are well-characterized, immunological processes triggering and maintaining this phenomenon are still poorly understood. As a consequence, no biotherapy targeting cytokines are currently efficient to treat airway remodeling and only bronchial thermoplasty may have an effect on bronchial nerves and smooth muscles with uncertain clinical relevance. Th17 cytokines, including interleukin (IL)-17 and IL-22, play a role in neutrophilic inflammation in severe asthma and may be involved in airway remodeling. Indeed, IL-17 is increased in sputum from severe asthmatic patients, induces the expression of “profibrotic” cytokines by epithelial, endothelial cells and fibroblasts, and provokes human airway smooth muscle cell migration in *in vitro* studies. IL-22 is also increased in asthmatic samples, promotes myofibroblast differentiation, epithelial-mesenchymal transition and proliferation and migration of smooth muscle cells *in vitro*. Accordingly, we also found high levels of IL-17 and IL-22 in a mouse model of dog-allergen induced asthma characterized by a strong airway remodeling. Clinical trials found no effect of therapy targeting IL-17 in an unselected population of asthmatic patients but showed a potential benefit in a sub-population of patients exhibiting a high level of airway reversibility, suggesting a potential role on airway remodeling. Anti-IL-22 therapies have not been evaluated in asthma yet but were demonstrated efficient in severe atopic dermatitis including an effect on skin remodeling. In this review, we will address the role of Th17 cytokines in airway remodeling through data from *in vitro, in vivo* and translational studies, and examine the potential place of Th17-targeting therapies in the treatment of asthma with airway remodeling.

## Introduction

Asthma is a chronic respiratory disease characterized by lower airway inflammation, leading to airway hyperresponsiveness (AHR) and airway obstruction, resulting in recurrent symptoms (chest wheezing, cough, dyspnea), exacerbations (flare-up) and altered quality of life. As one of the most frequent chronic respiratory disease, asthma is estimated to affect around 5–10% of adults (up to 20% in high-income countries) and from 5 to 20% of children. As a consequence, asthma is a major public health issue (1–4). The cornerstone of the treatment of asthma is inhaled steroids (ICS). Severe asthma is defined as asthma requiring at least high doses of ICS in association with another controller (generally long-acting beta-agonist bronchodilators) and/or oral steroids more than half a year to be controlled, or that remains uncontrolled despite these therapies (5–7). The burden of asthma is particularly heavy in patients with severe asthma who experience a severely impaired quality of life, respiratory disability, frequent and severe exacerbations, poorer control of symptoms, and hospitalisations. Severe asthma is also associated with lower respiratory function with persistent airway obstruction, representing about 50% of severe asthmatic patients, and sometimes loss of reversibility of airway obstruction (6–8). These patients account for the major part of asthma-related healthcare cost (6, 7).

From a pathophysiological point of view, persistent airway obstruction and loss of reversibility are the clinical hallmark of airway remodeling. Airway remodeling is a complex biological process, found in most chronic inflammatory airway diseases, which contributes to airway obstruction (9). In asthma, airway remodeling is characterized by several elementary structural changes in bronchial mucosa: loss of epithelial integrity, epithelial-mesenchymal transition (EMT), goblet cell hyperplasia, mucus hypersecretion, subepithelial fibrosis with thickening of epithelial basement membrane, smooth muscle cell proliferation, and hypertrophy. Inflammatory cell infiltration in bronchial mucosa can also be associated with airway remodeling and contribute to this process (10–12). These changes lead to increased lower airway resistances and reduced bronchial lumen, thus contributing to persistent airway obstruction. Although airway remodeling is present in asthma in all degrees of severity, airway remodeling intensity correlates with asthma severity (11, 13–17).

Asthma has long been considered as an allergic Th2-driven inflammatory disease, involving the “canonical” Th2 cytokines [interleukines (IL)-4, IL-5, IL-13], eosinophilic inflammation, and mediators of allergic inflammation (mainly immunoglobulins E and mast cells) (18). Over the last 20 years, major advances have been made in the understanding of asthma immunobiology and heterogeneity. Cluster analyses of large cohorts of patients allowed to identify several homogeneous subpopulations of asthmatic patients according to clinical characteristics (phenotypes), and their correlates in inflammatory and immunobiological processes (endotypes) (18–20). Hence, asthmatic patients are currently categorized according to the dominant inflammatory pathway driving bronchial inflammation, and particularly the degree of involvement of T2 inflammation: T2^high^ asthma (typically allergic and non-allergic eosinophilic asthma) vs. T2^low^ asthma (21). T2^low^ asthma refers to a heterogeneous group of patients with distinct endotypes, currently poorly characterized, like neutrophilic or paucigranulocytic inflammation.

Both patients with T2^high^ or T2^low^ asthma can present severe asthma. However, the neutrophilic endotype, characterized by a predominance of neutrophils in airway inflammation, has been particularly associated with severe asthma and resistance to ICS and oral steroids (22–25). In contrast, T2^high^ asthma is more commonly associated with response to steroids. Additionally, patients with T2^high^ severe asthma have now access to effective biotherapies that have emerged over the last decade, based on monoclonal antibodies targeting Th2 cytokines and immunoglobulins E (26). Although involvement of non-T2 pathways in asthma remains not fully understood, recent works have highlighted the role of Th17-driven inflammation in T2^low^ asthma (27–29). Th17 CD4^+^ cells produce IL-17 and IL-22, two key cytokines involved in neutrophilic inflammation in the context of antimicrobial host defense, and in the regulation of epithelial function and repairing. These cytokines are both also implicated in inflammatory disease pathogeny such as rheumatoid arthritis or psoriasis (30). As neutrophilic inflammation has been associated with T2^low^ asthma and severe asthma (22, 23), a role for Th17-type cytokines in asthma and airway remodeling has been rapidly postulated and gradually investigated over the past few years.

In this review, after going over the molecular and cellular mechanisms of the Th17 pathway, we will further scrutinize the existing evidences of the involvement of the Th17-type cytokines in asthma and airway remodeling. We will also comprehensively focus on the biological clues involving IL-17 and IL-22 in each of the elementary pathological component of airway remodeling. Eventually, an updated overview of the perspectives of Th-17 targeted therapies in asthma will be provided.

## TH17-Cytokine Producing Cells and TH17 Differentiation

In the 2000's, a subpopulation of CD4^+^ T cells, distinct from Th1 et Th2 cells, and characterized by the production of IL-17A (also named IL-17), IL-17F and IL-22 was identified and named accordingly to these cytokines “Th17 cells” (31–35). Their role in general inflammatory pathophysiology and more specifically in respiratory diseases and asthma has rapidly been investigated and led to the concept of a “Th17 pathway” and Th17-type cytokines (IL-17, IL-17F, and IL-22). Alongside Th17 cells, other cell types were subsequently found to be sources of Th17-type cytokines. Moreover, a subtype of CD4^+^ T cells producing only IL-22 but not IL-17 (Th22 cells), was later discovered (36, 37). More recently, a specific subset of innate lymphoid cells (ILC), named ILC3, has emerged as another important local cellular source of IL-17 and IL-22, especially in asthma (38, 39). Eosinophils were also found to produce IL-17 in asthma (40). Other cell types have been shown to produce Th17-type cytokines but their contribution to asthma pathophysiology is not fully elucidated: γΔ T cells, CD8^+^ T cells, B cells, Natural Killer T cells (41–46). Of note, mixed cytokine phenotypes have been identified in asthmatic patients such as Th2/Th17 (co-expressing IL-4 and IL-17) and Th1/Th22 [co-expressing Interferon (IFN) and IL-22] profiles (47, 48). This might be of interest as *in vitro* data seem to indicate that IL-4 and IFN-g play a role in Th1/Th2 polarization of human bronchial epithelial cells and may contribute to airway remodeling particularly through IL-4-induced expression of RUNX2 in epithelial cells which stimulates TGFβ production (49).

**Figure 1:**
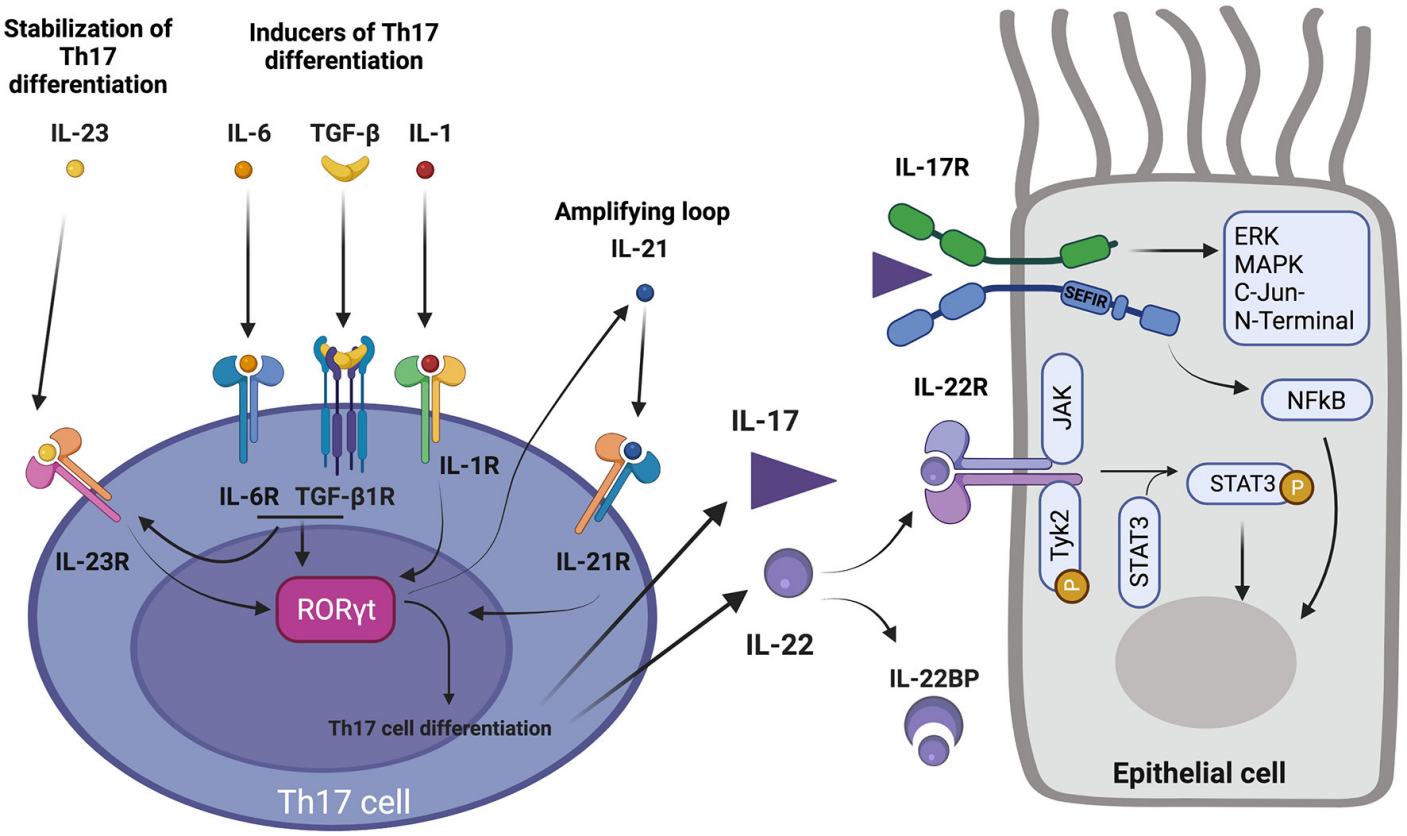
Th17 pathway. **(Left)** acquisition of Th17 differentiation. 1-IL-6 and TGFβ, and IL-1β promote RORγt expression and Th17 differentiation. 2-IL-6 and TGFβ induce IL-23R expression which allows IL-23 to stabilize Th17 differentiation. 3-Th17 differentiation also includes secretion of IL-21 which in turn enhances Th17 differentiation, acting as a positive feedback loop. 4- Secretion of IL-17 and IL-22 by Th17 cell. **(Right)** action of IL-17 and IL-22 on their respective receptors and activation of corresponding downstream pathways in an epithelial cell. 5- IL-22 binding protein (IL-22BP) regulates action of IL-22. 6-IL-22R activation induce Janus Kinase 1 (JAK) and Tyk2 associated kinases which lead to activation of transcription factor STAT3 by phosphorylation. 7-IL-17R binding by IL-17 activates SEFIR intracellular domain which activates ERK, MAPK, C-Jun N-terminal pathways, and transcription factor NF-κB.

Th17 differentiation is dependent on the high expression of the master transcription factor Retinoic acid receptor- related Orphan Receptor-γt (RORγt) (and to a lesser extent RORα), leading to Th17-type cytokine production (50, 51). Th17 differentiation is initially promoted either synergistically by IL-6 and TGF-β, or by IL-1ß, respectively through STAT3, SMADs, and AKT/mTOR and p38 pathway activations (52–61) (Figure 1). Aryl hydrocarbon receptor transcription factor ligands can also promote Th17 differentiation by binding their cytosolic receptor (62, 63). Amplifying auto-feedback loops involving IL-21 and IL-23 ensure stabilization of Th17 differentiation and proliferation of Th17 cells (44, 52). Of note, type 1 and 2 interferons, IL-27, and IL-4 cytokines have the ability to inhibit Th17 differentiation (64–67). Triggers of Th17 differentiation are multiple, including microbial environment (of which microbiome composition), sodium homeostasis, allergy, complement activation and inorganic particle exposure (61). Interestingly, in mice, the exposure to microbiota promotes the expansion of Th17 cells, ILC3, and regulatory T cells exhibiting characteristics of Th17 differentiation (expression of RORγt), which negatively regulates Th2 response, in intestinal mucosa (68). Th17 regular function is currently thought to promote tissue inflammation in order to ensure early clearance of extracellular pathogens for which Th1 and Th2 responses are insufficient (61, 69, 70). Th22 differentiation requires IL-6 and TNFa co-stimulation (Figure 1). IL-22 can also be co-secreted by Th1 and Th2 cells. Aryl hydrocarbon receptor has been suggested as the master transcriptional factor of Th22 cells but is not the only Th22 differentiation determinant (71).

## TH17 Cytokines and Pathways

IL-17A, initially known as CTLA-8, belongs to a family of cytokines including IL-17A, IL-17B, IL-17C, IL-17D, IL-17E (IL-25), and IL-17F. Among them, IL-17A (also known as IL-17) and IL-17F have been the most comprehensively studied members, and have been shown to play a physiological and pathophysiological role in humans (61). IL-17A mainly exists under a homodimeric form (72). IL-17A promotes inflammation through stimulation of granulopoiesis, induction of neutrophil-attractant and neutrophil-activating cytokines and chemokines (CXCL1, CXCL2, CXCL5, CXCL8, CXCL9, CXCL10, IL-6, G-CSF, and GM-CSF) (73–77). IL-17 effects are driven through its dimeric receptor IL-17R. In humans, IL-17R exists under several forms, of which IL-17RA and IL-17RC are respectively homodimeric cognate receptors for IL-17A and IL-17F. IL-17RA expression is ubiquitary (hematopoietic cells and structural non-hematopoietic cells like epithelial cells, endothelial cells, fibroblasts) conversely to IL-17RC, which is exclusively expressed in structural non-hematopoietic cells. Signaling *via* heterodimeric cytokine IL-17A/F and heterodimeric receptor IL-17RAC is also possible (78–80). IL-17 effects are transduced by the SEFIR intracellular domain of IL-17R. Downstream IL-17R signaling noticeably activates NFκB, ERK, and MAPK and c-Jun N-terminal kinase pathways (61) (Figure 1).

IL-22 belongs to the IL-10 cytokine family. It plays an important role in host defense in barrier tissues, and in epithelial protection and regeneration after injury (42, 46, 81–85). It enhances proliferation and migration of cells expressing IL-22R, while it inhibits their apoptosis and differentiation (86–89). The antimicrobial activity of IL-22 includes the stimulation of S100 protein and defensin productions (90). Interestingly, IL-22 production is dependent of the Aryl hydrocarbon receptor transcription factor (62, 91). IL-22 binds a heterodimeric receptor made up of the IL-22R1 and IL-10R2 subunits. Interestingly, IL-22R is only expressed on structural cells (epithelial and endothelial cells, fibroblasts, and smooth muscle cells) and not in hematopoietic cells. This repartition of IL-22R is highly relevant regarding the potential role of IL-22 in airway remodeling as these cell types are involved in airway remodeling processes. IL-22R mainly activates downstream STAT3 through transduction signals involving the receptor associated Janus kinases Jak1 and Tyk2 (92–94) (Figure 1). A soluble receptor for IL-22 [IL-22 binding protein (IL-22BP)], sharing high homology with IL-22R, contributes to IL-22 pathway regulation (95). IL-22 also plays a role in the tissue recruitment of neutrophils by inducing the production of neutrophil attracting chemokines, notably that of CXCL1 and CXCL5 by bronchial epithelial cells (96).

Interactions between IL-17 and IL-22 remain mostly not well-understood. Initially thought to be exclusively part of the same immunological response because of their common cellular source (Th17 cells, ILC3), each of these cytokines is also produced independently by many other cells (Th1 cells, Th22 cells, mixed Th1/Th22 cells for IL-22; Th2/Th17 for IL-17) (42, 48). Several studies suggest a mutual ability of IL-17 and IL-22 to inhibit each other production and respective Th17 and Th22 differentiation (97–99).

Beyond the homeostatic functions of IL-17 and IL-22, Th17 response is known to be involved in several chronic inflammatory diseases such as rheumatoid arthritis, psoriasis, psoriatic arthritis, ankylosing spondylitis, Crohn's disease, and atopic dermatitis (100–103). Among these diseases, some are particularly characterized by neutrophilic inflammation and tissue remodeling, such as psoriasis and atopic dermatitis. As a consequence, the role of IL-17 and IL-22 in asthma, has been questioned over the last two decades.

## Involvement of IL-17 and IL-22 in Asthma

The contribution of IL-17 in asthma was first suspected from the identification of asthmatic patients with neutrophilic inflammation in sputum as a distinct cluster of non-eosinophilic severe asthma (104, 105). Knowing the central role of IL-17 in neutrophil migration, recruitment and activation, multiple studies investigated the association between IL-17 levels in serum, sputum, bronchial biopsies and asthma. Most studies found not only increased IL-17 levels in asthmatic patients (28, 40, 106–108), but also positive correlation between IL-17 levels and asthma severity and association with airway neutrophilia (106, 109, 110). The presence of cells expressing IL-17 (ILC3 cells, Th17 cells) was also positively correlated with asthma severity (22, 39). Interestingly, specific Th2/Th17 CD4^+^ T cells, producing both Th2 and Th17 cytokines were identified in bronchoalveolar lavage (BAL) of asthmatic patients. Severe asthma was associated with increased Th2/Th17 cells predominantly polarized toward IL-17 production (Th2/Th17^high^) in BAL (111). The involvement of IL-17 in asthma pathogenesis was further confirmed in murine models, which globally found that ovalbumin sensitization (inhaled, intraperitoneal, or epicutaneous route) followed by inhaled challenge was associated with Th17 response, up-regulation of IL-17 in airways, and that up-regulation of IL-17 enhanced airway neutrophilia and steroid-resistant AHR (112–115). IL-17 production is also increased in obesity-associated murine models of asthma and asthmatic pediatric patients (38, 39, 116). Data from murine models are summarized in Table 1.

**Table 1:** Summary of data about the role of IL-17 in asthma from murine models.

**Study**	**Murine Model**	**Allergen**	**Route of sensitization**	**Method to study IL-17 effects**	**Phase of allergic inflammation investigated**	**Global effect of IL-17**	**Effect of IL-17 on Th2 cytokines**	**Effect of IL-17 on IL-22**	**Effect on eosinophilic inflammation**	**Effect on neutrophilic inflammation**	**Effect on AHR**	**Effect on airway remodeling**
Hellings et al. (117)	Balb/c	OVA	Ip	Anti-IL-17 Ab	Se+Chal	Pro-inflammatory	↓		NS	↑	↑	
He et al. (113)	Balb/c	OVA	•Ip •Ec	Anti-IL-17 Ab	Se+Chal	Pro-inflammatory	NS		NS	↑	↑	
McKinley et al. (115)	Severe combined immunodeficient Balb/c	OVA	N/A	•IL-17RA KO •Infection with IL-17 overexpressing Adenovirus	Chal	Pro-inflammatory			NS	↑	↑	
Wilson et al. (112)	C57BL/6	OVA	•Ip •It	IL-17RA KO	Se+Chal	Pro-inflammatory	NST		↓	↑	↑	NS (mucus)
Wang et al. (118)	C57BL/6	OVA	Ip	Anti-IL-17 Ab	Se+Chal	Pro-inflammatory				↑		↑ (mucus, s.e. fibrosis, SM hypertrophy)
Ano et al. (114)	Transgenic C57BL/6 (RORγt overexpression)	OVA	Sc	Anti-IL-17 Ab	Se+Chal	Pro-inflammatory			NS	↑	↑	
Zhao et al. (119)	C57BL/6	OVA	•Ip •It	Stimulation with IL-17	Se+Chal	Pro-inflammatory			↑	NS		↑ (alteration of epithelial integrity)
Kim et al. (39)	C57BL/6 + High Fat Diet	N/A	N/A	IL-17 KO	N/A	Pro-inflammatory					↑	
Camargo et al. (120)	Balb/c	OVA	Ip	Anti-IL-17 Ab	Se+Chal	Pro-inflammatory	↑		↑	↑		↑ (s.e. fibrosis, MMP-9 production)
Lamb et al. (108)	Balb/c	N/A	N/A	•Stimulation with IL-17 •Anti-IL-17 Ab	N/A	Pro-inflammatory		NS	NS	NS	NS	

*OVA, ovalbumin; Ip, intraperitoneal; Sc, subcutaneous; Ec, epicutaneous; It, intratracheal; Ab, antibody; KO, knock out gene; Se, allergen sensitization; Chal, allergen challenge; NST, effect of IL-17 non-specifically tested; NS, non-significant effect; AHR, airway hyperresponsiveness; s.e, subepithelial; SM, smooth muscle; MMP-9, matrix metallopeptidase 9; N/A, non-applicable*.

Similarly, IL-22 and IL-22R upregulation was suggested in several studies in serum and in bronchial biopsies from asthmatic adults and children (predominantly in severe asthmatic patients) and in murine models of asthma, as well as in patients with other allergic respiratory diseases such as atopic rhinitis (99, 121–124). IL-22 producing cells, particularly lymphocytes, are also increased in bronchial biopsies from asthmatic children (123).

However, the role of IL-22 as a protective or a pathogenic actor in asthma remains less clear than that of IL-17, as pathogenic effect failed to be constantly reproduced in murine models. Indeed, conflicting evidences have shown that IL-22 could have deleterious but also beneficial effects on airway epithelium in asthma (42, 95, 125, 126) (Table 2). Hence, a protective action of IL-22 on airway allergic inflammation, in particular on airway eosinophilic inflammation and Th2 cytokine production, on AHR and even on some pathological feature of airway remodeling (goblet cell hyperplasia) has been described in several studies (127–129, 131). On the other hand, as underlined by Hirose et al. in an extensive review about dual effects of IL-22 on airway epithelium, atopic dermatitis models and some murine models of asthma found pro-inflammatory effects of IL-22, with enhanced AHR and airway remodeling (mucus hyperproduction and goblet cell hyperplasia, and smooth muscle cell hyperplasia) (95, 99, 133, 134). These contrasting results have been discussed in a previous review (125) (Table 2). One of the main differences between these studies is the route of sensitization, which may account for a differential effect of IL-22 with a protective effect associated with the intraperitoneal route, and pro-inflammatory effects associated with the subcutaneous route. Additionally, the protective or pro-inflammatory effects of IL-22 were differently exerted according to the phase of the allergic reaction (sensitization or challenge). In this regard, the work of Leyva-Castillo et al., which tried to mimick the atopic march, is interesting. They found that epicutaneous sensitization with ovalbumin in mice further challenged with intranasal ovalbumin led to increased expression of IL-22 in serum and lungs, which was associated with enhanced AHR and mixed neutrophilic and eosinophilic airway inflammation. This was mainly observed with epicutaneous sensitization and not intraperitoneal sensitization (132). Overall, the precise role of IL-22 in asthma is still unclear, probably dependent on many parameters, such as the timing of action during the allergic process, the route of sensitization, as well as the immunological microenvironment regulating the Th22 response (Table 2). One can hypothesize that when IL-22 response is imbalanced, its physiological role may turn into a harmful action.

**Table 2:** Summary of studies showing conflicting evidence about the role of IL-22 in asthma murine models.

**Study**	**Murine Model**	**Allergen**	**Route of sensitization**	**Method to study IL-22 effects**	**Phase of allergic inflammation investigated**	**Global effect of IL-22**	**Effect of IL-22 on Th2 cytokines**	**Effect of IL-22 on IL-17**	**Effect on eosinophilic inflammation**	**Effect on neutrophilic inflammation**	**Effect on AHR**	**Effect on airway remodeling**
Takahashi et al. (127)	Balb/c	OVA	Ip	Anti-IL22 Ab	Se+Chal	Protective			↓		↓	
Taube et al. (128)	C57BL/6	OVA	Ip	IL-22 KO	•Se+Chal •Chal wo Se	Protective during Se			↓		↓	
Nakagome et al. (129)	Balb/c	OVA	Ip	•IL-22BP •Inducible-IL-22 expression	•Se •Se+Chal	Protective during Se			↓ only during Se			↓ (mucus) only during Se
Besnard et al. (99)	C57BL/6	OVA	Sc	•Anti-IL22 Ab •IL-22 KO	•Se •Se+Chal	•**Pro-inflammatory** during Se •Protective during Chal	↑ only during Se	↓	↑ only during Se		↑ only during Se	↑ (mucus) only during Se
Fang et al. (130)	C57BL/6	OVA	Ip	•Lung-inducible IL-22 expression	Se+Chal	Protective	↓		↓		↓	↓ (mucus)
Ito et al. (131)	C57BL/6	HDM	Intratracheal	IL-22 KO	Se+Chal	Protective			↓	↓	↓	
Leyva-Castillo et al. (132)	Balb/c	OVA	•Ec •Ip	•Anti-IL22 Ab •IL-22 KO	Se+Chal	**Pro-inflammatory** with Ec Se			↑ (only after Ec Se)	↑ (requires TNFα) only after Ec Se)	↑ only after Ec Se	

*OVA, ovalbumin; HDM, house dust mite; Ip, intraperitoneal; Sc, subcutaneous; Ec, epicutaneous; Ab, antibody; KO, knock out gene; IL-22BP, IL-22 Binding Protein; Se, allergen sensitization; Chal, allergen challenge; wo, without; AHR, airway hyperresponsiveness*.

Of interest, several studies have shown a global stimulation of the Th17 response in asthma, with simultaneously increased expression of IL-17 and IL-22 in peripheral mononuclear blood cells (PMBC) and bronchial biopsies from asthmatic patients, this increase being even higher in severe asthmatic patients. Furthermore, increased levels of IL-17 and IL-22 were insensitive to steroids, a characteristic frequently found in patients with neutrophilic airway inflammation and with airway remodeling and specific to severe asthma (25, 116, 135, 136). In a recent work, our group also reported airway remodeling and neutrophilia in a murine model of allergic asthma induced by dog allergen. In this model, IL-17 and IL-22 expression were both upregulated (137).

In the era of “Omics,” two studies looked at transcriptomics in blood, sputum and bronchial biopsies of asthmatic patients, mainly in the U-BIOPRED cohort. Thereby, Badi et al. identified in asthmatic patients a transcriptomic signature previously identified in atopic dermatitis, a well-known Th22-driven inflammatory disease. This signature included upregulation of Th17 and Th22 pathway-related genes. Enrichment of this signature in biological samples from asthmatic patients was positively correlated with asthma severity (138). Similarly, Lamb et al. found an upregulated Th17 transcriptomic signature in bronchial biopsies from asthmatic patients (108).

Finally, both IL-17 and IL-22 levels have been found to be negatively correlated with FEV_1_ (25, 136, 139), a hallmark of severe asthma with airway remodeling. Overall, the involvement of IL-17 in asthma is well-established. Although the involvement of IL-22 in asthma seems very likely, its exact role is still to be clarified.

## IL-17, IL-22, and Neutrophilic Inflammation in Asthma

Airway inflammation contributes to the airway remodeling process (10). In asthma, airway inflammation is usually characterized by T2 inflammation with eosinophilic cell infiltration of airways. Nevertheless, airway neutrophilia is classically associated with severe asthma (23). Neutrophilic asthma is also associated with steroid resistance, poorer lung function (FEV_1_) and lower reversibility of airway obstruction, which can be accounted by airway remodeling (22, 25, 140–142).

IL-17 and IL-22 are involved in neutrophilic inflammation in asthma. As demonstrated on bronchial biopsies from a large cohort of asthmatic patients, airway neutrophilia is associated with both increased IL-17 and IL-22-producing cells (22, 108). More recently, Badi et al. showed in a cohort of asthmatic patients that the degree of enrichment in atopic dermatitis-similar transcriptomic signature, including overexpression of Th17 and Th22-related genes, was strongly associated with severe neutrophilic asthma (138).

Indeed, IL-17 is a key cytokine in the recruitment and activation of neutrophils (69, 76). Neutrophilia is correlated with IL-17 expression in airway epithelium (108). In murine models of asthma, IL-17 stimulates and orchestrates airway neutrophilic infiltration by inducing the expression of neutrophil-attractant chemokines like CXCL1, CXCL2, IL-6, and IL-8 in humans (75, 108, 112–115, 117, 143).

Though its place still needs to be clarified in airway neutrophilia, IL-22 seems also to be an important factor in neutrophil recruitment (144, 145). It is able to also enhance production of neutrophil recruitment associated chemokines like CXCL8 (in humans), CXCL1, CXCL2, CXCL3, CXCL5, and IL-1b, particularly in mold allergen-induced asthma murine models (132, 137, 143, 144, 146). Interestingly, several data suggest a pro-inflammatory effect of IL-22 synergistically with IL-17 on airway neutrophilia by inducing the expression of chemokines by epithelial cells but also by airway smooth muscle cells (98, 108, 143, 147).

Overall, the association between both of these cytokines and neutrophilic inflammation is highly relevant, knowing that neutrophils are thought to be an effector cell of airway remodeling, particularly through the release of proteolytic enzymes like elastases and metalloproteases (notably MMP-9), thus participating in structural change of the extracellular matrix of bronchial epithelium (148, 149).

## Involvement of IL-17 and IL-22 in Airway Remodeling

Experimental data provides substantial arguments for an involvement of IL-17 and IL-22 in airway remodeling through their contribution to the diverse pathobiological processes that lead to airway remodeling: subepithelial fibrosis, mucus hyperproduction, airway inflammation and smooth muscle cell hyperplasia and hypertrophia.

Out of the field of asthma, IL-17 and IL-22 enhance airway remodeling in murine models of lung injury by pollutants (airway neutrophilic infiltration, epithelial desquamation, subepithelial fibrosis) (150). They are also involved in tissue remodeling in other inflammatory diseases (bone remodeling in psoriatic arthritis, skin remodeling in atopic dermatitis) as well as in other chronic respiratory diseases, such as chronic obstructive pulmonary disease (COPD) (76, 100, 151–158).

The enhancement of airway remodeling by IL-17 has been confirmed in murine models of asthma in which its blockade by anti-IL-17 antibodies or genetic deficiency reduced mucus hypersecretion, goblet cell hyperplasia, subepithelial collagen deposition and airway smooth muscle layer thickening together with airway neutrophilia (118, 119). Interestingly, IL-17 seemed to increase epithelium permeability to allergen, indicating a loss of epithelial integrity, also observed in airway remodeling (119). IL-17-induced airway remodeling seemed to be dependent on IL-6 (119), and to be partially mediated through heparin-binding epidermal growth factor (HB-EGF) (118).

Regarding the specific topic of airway remodeling, the understanding of the role of IL-22 suffers from conflicting evidences. Fang et al. found a modulatory effect of IL-22 with decreased eosinophilic inflammation and Th2 cytokine secretion, decreased AHR but also reduced mucus production and goblet cell hyperplasia in lung-specific IL-22 transgenic model of ovalbumin-induced asthma (130). Paradoxically, in another murine model of dog allergen-induced asthma, which reproduces the characteristics of airway remodeling, Aryl hydrocarbon receptor antagonism decreased IL-22 levels (but not IL-17) in lungs together with decreased airway remodeling. The reduction of subepithelial fibrosis, mucus hyperproduction and smooth muscle thickness after treatment with Aryl hydrocarbon receptor antagonist suggests an association between airway remodeling and IL-22 (137). Interestingly, as previously underlined, IL-22R is not expressed on hematopoietic cells but only on structural cells (epithelial, endothelial cells, fibroblasts, smooth muscle cells) which are major contributors of airway remodeling (42).

Interestingly, B cells, particularly regulatory B cells, seem to have a beneficial regulatory effect on AHR and airway remodeling in a murine model of asthma (159). Although there is no specific data on the role of Th17 cytokines in this regulatory process, several works have showed that Th17 cells, IL-17 and IL-22 (though this is an indirect effect as B cells do not express IL-22R) may exert an important role of recruitment, stimulation and maturation of B cells and a regulatory role of B cell response in mucosal barriers (160–166). Subsequently, Th17 cytokines might also contribute to airway remodeling by an indirect effect of regulation of B cell response.

Respiratory tract infections, and in particular viral infections, may also contribute to the pathophysiology of asthma and the development of airway remodeling as a bronchial chronic inflammation trigger, especially in T2^low^ asthma (167–169). In this setting, knowing the role of IL-17 and IL-22 in antimicrobial host defenses in mucosal barriers (170–172), infections may play an important role of trigger of Th17-induced airway remodeling in patients with recurrent infection phenotype, by inducing chronically high levels of Th17 cytokines in lungs.

**Figure 2:**
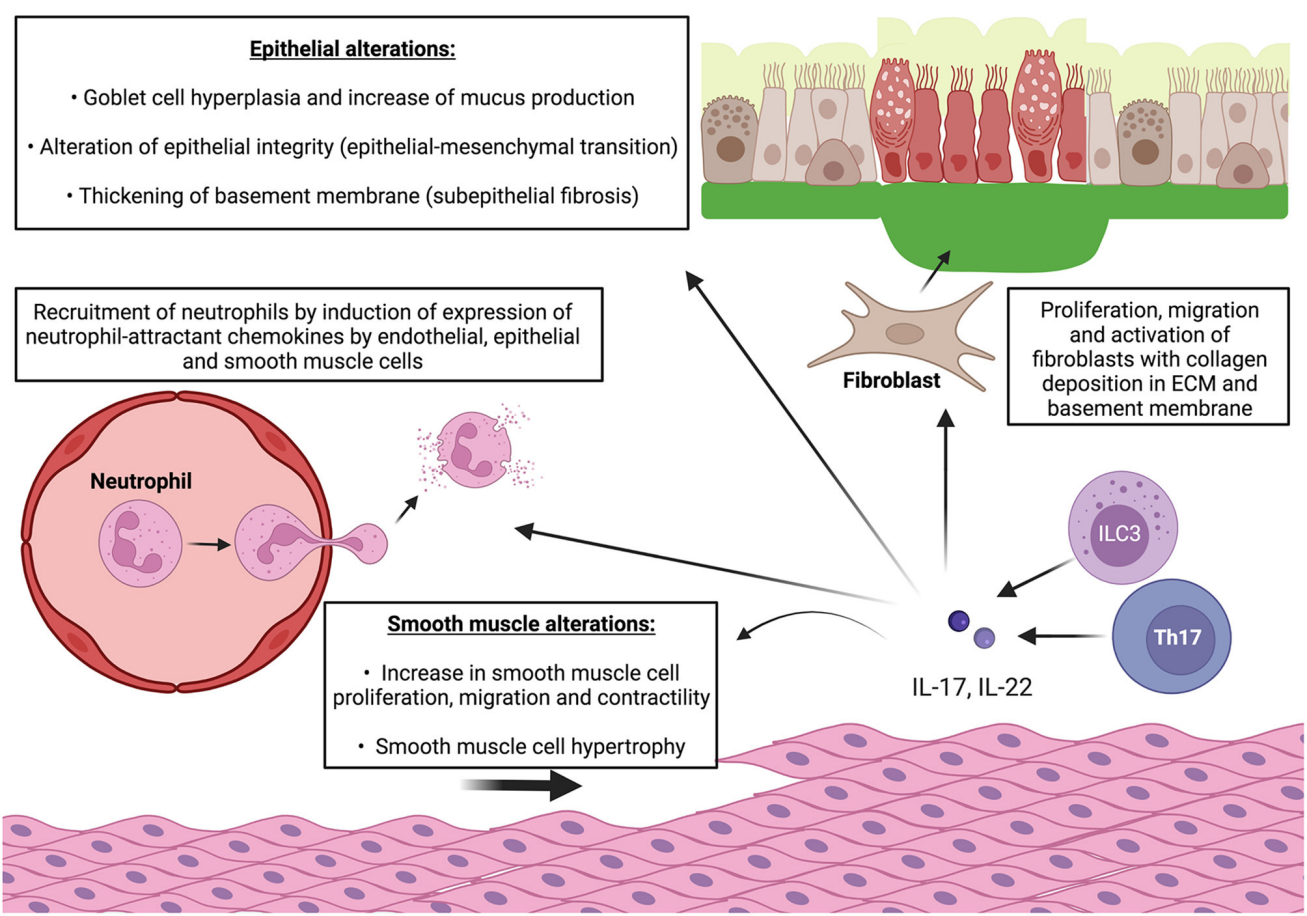
IL-17 and IL-22 cooperate to induce features of airway remodeling in asthma. ECM: extracellular matrix. Pro-inflammatory effect: release of IL-17 and IL-22 by Th17 cells and ILC-3 induce expression of neutrophil-attractant chemokines by endothelial cells and enhance neutrophil recruitment and diapedesis. IL-17 and IL-22 also induce expression of neutrophil-attractant and neutrophil-activating chemokines by epithelial cells and smooth muscle cells which lead to local neutrophilic inflammation with release of proteolytic enzymes (elastase, metalloproteases). Epithelial alterations: IL-17 and IL-22 also increase production of mucus by goblet cells and promotes goblet cell hyperplasia. They also alter epithelial integrity by enhancing E/N-cadherin switch which leads to epithelial-mesenchymal transition. Smooth muscle alterations: IL-17 and IL-22 promote smooth muscle cell proliferation, migration, and contractility (only IL-17). Subepithelial fibrosis: IL-17 and IL-22 stimulate fibroblast proliferation and switch to pro-fibrotic phenotype and promote collagen production and deposition in ECM relsulting in thickening of basement membrane. IL-22 also promotes migration of myofibroblast in smooth muscle cell bundles which contributes to local fibrosis and smooth muscle contractility.

Overall, IL-17 involvement in airway remodeling seems well-established while it is less clear for IL-22. However, *in vitro* data provide numerous clues in favor of the involvement of both of these cytokines in several biological processes contributing to airway remodeling (Figure 2).

### Subepithelial Fibrosis

Subepithelial fibrosis is a major feature of airway remodeling in asthma. It is mainly characterized by increased collagen deposition in the extracellular matrix (ECM), resulting in thickening of basement epithelial membrane of bronchial mucosa and reduced airway compliance. This phenomenon contributes to airway obstruction. Mainly fibroblasts, but also smooth muscle cells are the cellular effectors of subepithelial fibrosis in asthma. TGF-β and IL-11 are the main profibrotic cytokines which stimulate collagen secretion by fibroblasts (9–12).

In chronic inflammatory bowel diseases, the effect of Th17-type cytokines on subepithelial fibrosis through their action on myofibroblasts is well-described (173). If Th2-type cytokines IL-4 and IL-13 involvement in subepithelial fibrosis in asthma is acknowledged (174, 175), pro-fibrotic effects of IL-17 are also well-documented. Indeed, IL-17 induces the production of pro-fibrotic cytokines IL-6 and IL-11 by fibroblasts obtained from bronchial biopsies of asthmatic patients (40). Additionally, Bellini et al. showed that circulating fibrocytes isolated from exacerbating asthmatic patients had increased expression of IL-17RA as compared to fibrocytes from control healthy subjects (175). Both IL-17A and IL-17F (but not Th2 cytokines) have demonstrated the ability to induce release of pro-neutrophilic chemokines (CXCL1, CXCL8, and TNFa) by fibrocytes, and to enhance fibrocytes switching toward a profibrotic phenotype (increased production of α-smooth muscle actin (SMA) mediated by CXCL8, of collagen, and of proangiogenic factors like VEGF and angiogenin) and fibrocyte proliferation (175, 176). This proliferative effect could be mediated by IL-6 and the Leukemia inhibitory factor (LIF), which were both found increased after IL-17 stimulation in this model (175). Al-Muhsen et al. suggested that eosinophils could also contribute to the IL-17-driven subepithelial fibrosis process. *In vitro*, the stimulation of eosinophils from asthmatic patients by IL-17 (but not by Th1 nor Th2 cytokines) induces the expression of the pro-fibrotic cytokines IL-11 and TGF-β1. However, this effect requires a co-stimulation with both IL-17A and IL-17F and can be synergistically enhanced by co-stimulation with IL-23, a known inducer and upregulator of IL-17R. This effect is driven by the p38 MAPKinase pathway (177). More recently, an original work highlighted that IL-17 induces autophagy and mitochondrial dysfunction in human fibroblasts from asthmatic patients, associated with impaired apoptosis and increased pro-fibrotic response (178).

Similar data suggests an involvement of IL-22 in subepithelial fibrosis in asthma. Liu et al. showed that blood from asthmatic children containing high levels of IL-22 and IL-22-producing lymphocytes had the ability to stimulate embryonic fibroblast proliferation and production by fibroblasts of collagen (α-1 and α-2 chains of collagen 1), thus suggesting a contribution of IL-22 to subepithelial fibrosis in asthma through its action on fibroblasts. This effect is linked to the activation of JAK/STAT3 signaling pathway by IL-22R1 (123).

Moreover, fibroblasts from bronchial biopsies of asthmatic patients have also been shown to promote Th17 differentiation of naïve CD4^+^ lymphocytes and secretion of IL-17 and IL-22, which in turn stimulates the production of pro-fibrotic cytokines (IL-6, TGFb) by fibroblasts but also cytokines favoring Th17 differentiation (IL-23, IL-6, and IL-1b). This suggests that the microenvironment can also promote a Th17 response in asthmatic patients, through the existence of amplifying loops in the fibrosing processes (179).

Subepithelial fibrosis also requires ECM remodeling involving proteolytic activity. In this setting, several studies interestingly showed, in murine models of asthma exacerbation, that IL-17 enhanced the production of metalloproteases MMP-9 and MMP-12, neutrophil elastase, and the myeloperoxidase activity, as well as ECM collagen deposition (120, 180).

Finally, biopsies from moderate to severe asthmatic patients showed increased expression of IL-17 in epithelial and specifically in subepithelial compartments, the latter being the location of subepithelial fibrosis, thus arguing in favor of a likely involvement of IL-17 in subepithelial fibrosis in humans. However, downregulation of IL-17 by a 2 week-course of oral steroids did not affect collagen deposition (181).

Taken together, these data suggest that IL-17 and IL-22 can promote subepithelial fibrosis in asthma by a direct action on fibrocytes and fibroblasts, activating collagen production and deposition in ECM, promoting metalloprotease activity, angiogenesis and cell proliferation, enhancing Th17 response and *in situ* neutrophil recruitment, and inhibiting fibroblasts apoptosis.

### Mucus Hyperproduction and Goblet Cell Hyperplasia

Another critical aspect of airway remodeling is represented by changes in mucus secretion function. From a pathological point of view, in asthma, airway remodeling is associated with goblet cell hyperplasia/metaplasia and mucus hypersecretion. From a biochemical point of view, changes in mucus rheology and composition are also observed (9–12).

Normal mucus composition is dominated by mucin glycoproteins, of which MUC5AC and MUC5B are the most abundant. It has been demonstrated *in vitro* that IL-17 (but not Th2 cytokines) was able to increase *MUC5AC* and *MUC5B* expression by bronchial epithelial cells (182–184). Stimulation of *MUC5AC* production is dependent on NfκB transcription factor (183). IL-17 also induces IL-6 production through activation of the JAK2 pathway, which in turn activates the ERK pathway, responsible for enhancement of MUC5B production (182).

At the tissue-scale, IL-17 stimulation increases the number of goblet cells which are the cellular source of mucus in airways (185, 186). Moreover, a recent work seems to implicate IL-17 in goblet cell metaplasia (187).

In addition, a chronic exposition to Aspergillus-induced model of allergic asthma in mice showed features of airway remodeling, noticeably airway inflammation and goblet cell hyperplasia with mucus hyperproduction, associated with lung function alteration. Dectin-1 is a C-type lectin receptor (CLR) that is primarily responsible for β-glucan recognition and control of fungal infection. Interestingly, the features of asthma in this model of chronic exposure to aspergillus were reduced by Dectin-1 deficiency which also downregulated IL-17 and IL-22 production. Of note, neutralization of IL-22 improved lung function in this model. These data suggests that IL-22 could be also involved in alteration of mucus secretion in asthma (146).

### Epithelial-Mesenchymal Transition (EMT) and Epithelial Alterations

Epithelial structure alteration is a key component of airway remodeling. In this process, most structural changes lead to altered epithelium integrity, epithelial cell damage and apoptosis, as well as increased epithelial permeability. Among these changes, a phenotypic switch to mesenchymal cell characteristics is observed in epithelial cells, thus achieving the epithelial-mensenchymal transition (EMT). One of the most critical changes concerns junction proteins, with loss of intercellular tight junction, especially downregulation of the expression of E-cadherin, leading to increase in epithelial permeability (9–12). Additionally, acquisition of a mesenchymal phenotype is likely to contribute to airway fibrosis (188).

In this setting, a potential role for IL-22 is very likely regarding its role in protection, restauration and maintenance of epithelial barrier homeostasis (125, 156). *In vitro*, TGF-β1-induced EMT in primary epithelial bronchial cells of severe asthmatic patients is enhanced by addition of IL-22. IL-22-enhanced EMT is characterized by acquisition of morphologic characteristics of mesenchymal cells (“spindle cells”), further decrease in E-cadherin expression and further increase in N-cadherin expression. This effect seems to be driven by Zeb1 transcription factor (121).

IL-17 has been involved in EMT in various respiratory diseases, like idiopathic pulmonary fibrosis, lung cancer, or COPD (189–192). In a translational work on obliterans bronchiolitis, Vittal et al. found that IL-17 could induce EMT, partially through TGF-β production, resulting in decreased expression of E-cadherin (193). In *in vitro* models, IL-17 induces, synergistically with IL-4 and TGF-β, reentering in cell cycle (proliferation) of epithelial cells, fibroblast morphotype switch, downregulation of E-cadherin, and upregulation of α-SMA (157, 194). A synergistic effect of cigarette smoke together with IL-17 on EMT was also reported by Ma et al. (191).

Interestingly, neutrophils from asthmatic patients were recently shown to have the ability to induce EMT (195). Given the preponderant role of IL-17 and IL-22 in neutrophil recruitment, this supports the involvement of both of these cytokines in EMT in asthma.

### Increase of Airway Smooth Muscle Mass

Increase of airway smooth muscle mass participates to airway obstruction in asthma and is one of the hallmark of airway remodeling (9–12). It results from several mechanisms including increased smooth muscle cell proliferation (hyperplasia), smooth muscle cell hypertrophy, abnormal migration properties leading to increased number of cells in smooth muscle bundles, especially myofibroblasts which also contributes to local fibrosis (12).

In *in vitro* models, IL-17A and IL-17F, as well as IL-22 promote airway smooth muscle cell (ASMC) proliferation, induce ASMC migration through activation of their respective cognate receptors and downstream activation of MAPKinases and NFκB pathways, and inhibit ASMC apoptosis (196, 197). IL-17-induced ASMC migration is also mediated by Growth-Related Oncogens (GRO), GROα being CXCL1, also involved in neutrophil migration (198). Neutrophils contribute to smooth muscle cell hyperplasia, which is critical given that IL-17 and IL-22 play an important role in neutrophil recruitment, particularly in airway smooth muscle bundles (199).

Additionally, translational data from two studies indicate that IL-17 but not IL-22 can enhance smooth muscle cell contraction *via* NF-κB/RhoA/ROCK2 signaling cascades, which are involved in regulation of phosphorylation of myosin light chain and thus ASMC contractility (200–202).

Overall, IL-17 and IL-22 stimulates ASMC proliferation and contractility, smooth muscle cell infiltration by neutrophils, migration of myofibroblasts into the smooth muscle layer, inhibit ASMC apoptosis, leading to thickening of airway smooth muscle mass.

## Therapeutic Directions

As they are tightly linked to asthma immunobiology, and especially to severe asthma, IL-17 and IL-22 appear as promising targets in the treatment of severe asthma. In regard to the overview of the very likely role of IL-17 and IL-22 in airway remodeling in asthma that we provide in this review, this therapeutic perspective is particularly interesting as there is currently no specific pharmacological treatment targeting airway remodeling in asthma, whereas several biotherapies are available for patients with severe T2^high^ asthma. To date, mainly bronchial thermoplasty, an interventional endoscopic procedure, offers a treatment option for these patients (203). Recently, tezepelumab, a human monoclonal antibody blocking thymic stromal lymphopoietin (TSLP), an epithelial-cell–derived cytokine which, as an alarmin, plays an early role in the inflammatory cascade in asthma, showed significant reduction of exacerbation rate in non-selected patients with severe asthma, including patients with non-T2 asthma. The effect was consistent in the subgroup of patients with low T2 biomarkers (low blood eosinophil count and low FeNO), suggesting that the effectiveness of this biotherapy is maintained in patients with non-T2 asthma (204). Of note a recent phase 2 clinical trial did not show any improvement on airway remodeling (epithelial integrity and basement membrane thickness) (205). Nevertheless, tezepelumab may offer a therapeutic option for patients with severe non-T2 asthma and could prevent the development of non-already established airway remodeling by early reduction of bronchial inflammation.

In the setting of Th17-targeting treatment of severe asthma, IL-17 blockade strategy has been the most studied so far (Table 3). According to the results obtained in the previously mentioned murine models of asthma, the effect of brodalumab, a humanized anti-IL17 monoclonal antibody already approved in the treatment of psoriasis, was investigated in a randomized controlled trial (RCT) in non-selected patients with moderate-to-severe asthma (206). No significant difference was found on the primary outcome criteria, which was the control of asthma based on the Asthma Control Questionnaire (ACQ). Nevertheless, the results of this study have been extensively discussed on two points. First, there was no selection of patients according to phenotype or endotype, and, as a consequence, many patients with T2^high^ asthma may have been included, a category of patients for whom a beneficial effect of anti-IL-17 treatment would be less expected than for patients with neutrophilic asthma. Second, the primary outcome criteria was improvement of asthma control, a criteria which has not been found improved by any other already-registered biotherapies in severe asthma, and which also may not reflect improvement in airway remodeling. Interestingly, a subgroup of patients with high FEV_1_ reversibility at inclusion demonstrated a significant difference in asthma control, suggesting that patients with not-yet-established permanent obstruction and airway remodeling might benefit from IL-17 blockade strategy in the early phase of airway remodeling (206, 209, 210). Recently, a phase I trial studying the effect of a bispecific anti-IL-13 and anti-IL-17A antibody in asthma was early terminated due to high frequency of treatment immunization (207). Additionally, experimental data suggest that blockade of IL-17 may restore sensitivity to steroids, including airway remodeling features (211). Lastly, a phase 2a RCT found that risankizumab, an anti-IL-23 monoclonal antibody, induced downregulation of Th17 associated transcription factors, and showed deleterious effects of outcomes (time to first worsening, annual rate of asthma worsening) in an unselected population of severe asthmatic patients (208). This result may put in perspective the protective aspects of Th17 response in asthma (anti-microbial defense in particular, although the proportion of infectious exacerbations was not specifically reported in this study) (209). Therefore, more data are needed on IL-17 blockade strategy. Trials selecting patients based on an “IL-17 phenotype” using biomarkers like sputum neutrophilia, IL-17 levels in blood or sputum, or airway remodeling biomarkers are expected to make progresses in this field.

**Table 3:** Summary of results from pre-clinical and clinical studies on Th17-related therapies.

**Drug**	**Biochemical nature and target**	**Type of study**	**Population of study**	**Primary endpoint**	**Effect on primary endpoint**	**Effect on asthma control**	**Effect on exacerbations**	**Effect on FEV1**	**Adverse events**	**Remark**
Brodalumab (206)	Human anti-IL-17RA IgG2 monoclonal Ab	Multicentric RCT, db, Ph3	18–65 y.o. moderate-to-severe asthmatics on stable ICS, no biomarker required (305 patients)	Asthma control (ACQ)	NS	NS		NS	Injection site reaction	Possible positive effect on asthma control in the high FEV1 reversibility subgroup
BITS7201A (207)	Humanized bispecific anti-IL-13 and -IL-17 IgG4 Ab	Monocentric RCT, ob, Ph1	Healhy volunteers (41 patients)	Pharmacokinetic parameters					Frequent development of anti-drug antibodies	
Risankizumab (208)	Humanized anti-IL-23 IgG1 monoclonal Ab	Multicentric RCT, db, Ph2	18–75 y.o. asthmatics on moderate ICS dose at least + 1 other controller, FEV1 40–85%, at least 2 severe exacerbations in the 12 last months (214 patients)	Time to first worsening:	Worsening (shorter time to first worsening with treatment)	NS	Worsening	NS	NS	Downregulation of Th17-associated transcriptome
BIX119 (108)	Small molecule, specific RORγt inhibitor	Pre-clinical (murine model)	Balb/c mice							Reduction of neutrophilic inflammation and AHR in mice
Tezepelumab (204)	Human anti-TSLP IgG2 monoclonal antibody	Multicentric RCT, db, Ph3	12–80 y.o. asthmatics on high ICS dose + 1 other controller, no biomarker (1,061 patients)	Annualized exacerbation rate	Improvement	Improvement	Improvement	Improvement	Headaches, Upper respiratory tract infections	

*RCT, randomized controlled trial; db, double blind; Ph, phase; y.o., year-old; NS, non-significant effect; AHR, airway hyperresponsiveness; ob, observer-blinded*.

Additionally, beyond the non-T2 asthma field, specific allergen immunotherapy in allergic rhinitis is associated with decreased serum levels of IL-17 mRNA and IL-17 as compared to non-treated controls (212, 213). Furthermore, the ratio between B regulatory cells (Breg) and Th17 cells (Breg/Th17 ratio) obtained from blood samples taken in the early phase of immunotherapy, positively correlates with a symptom score of response to this treatment at 3 years (214). This effect of immunotherapy on Th17 response might be driven through transdifferentiation of Th17 cells toward Treg cells, contributing to immunotolerance to allergens (215, 216). Interestingly, a recent work suggested that specific allergen immunotherapy-induced immunotolerance was associated with a decrease in “exhausted-like” Th2 cells in a murine model of chronic exposure to ovalbumin allergen and in patients with allergic rhinitis, a specific phenotype of Th2 cells characterized by the expression of CTLA-4 and PD-1 (217), which has paradoxically been proposed to contribute to the enhancement of Th2 response (218, 219). Of note, in their murine model, immunotherapy was associated with decreased neutrophil count and levels of IL-17 in BAL but increased expression of IL-17 and IL-22 in lung homogenates (217). Similarly, modulation of ILC2 exhausted phenotype might be a relevant target, particularly knowing their involvement in regulation of chronic allergic inflammation and that a subset of these cells can produce IL-17 (220, 221). Improvement in the understanding of the regulatory role of exhausted phenotype of T2 inflammation-associated cells in allergic inflammation and of their interaction with Th17 pathway may contribute to the development of therapies indirectly targeting Th17 pathway which may also impact Th17-associated airway remodeling.

Although there are no currently clinical studies of IL-22 targeting therapies in asthma, some data suggest a potential role for such therapies. First, the efficacy of the anti-IL-22 antibody fezakinumab was demonstrated in a randomized controlled trial in atopic dermatitis, a disease which shares many pathophysiological features with asthma and especially neutrophilic asthma (tissue remodeling, neutrophilic inflammation) (222). As previously mentioned, data on IL-22 blockade strategy from murine models provide conflicting evidence of the benefit of this strategy. Interestingly, Badi et al. found a predictive transcriptomic signature of response to fezakinumab in atopic dermatitis which was enriched in blood of patients with severe asthma from U-BIOPRED cohort. This enrichment was especially associated with neutrophilic asthma. The response to fezakinumab signature was correlated with Th22 related gene expression in blood and sputum (especially IL-22, CCR10, Aryl hydrocarbon receptor). IL-22 expression in sputum correlated with the enrichment of the response signature (138). Given the established role of IL-22 in epithelial physiology and in tissue remodeling (157), it might be an interesting target for the treatment of airway remodeling in asthma.

Finally, a new and potent RORγt inhibitor, administered in a mouse model of house dust mite-induced asthma which exhibited a transcriptomic signature overlapping with that of a cluster of patients with severe neutrophilic asthma from the U-BIOPRED cohort, showed a decrease in IL-22, IL-17, and CXCL1 levels in BAL fluid, in BAL neutrophilia and more interestingly an improvement in airway resistances in a dose dependent manner (108). This effect was not achieved by an anti-IL-17 antibody alone. This result may confirm the synergistic action of IL-17 and IL-22 that has been suggested in several studies (98, 108, 143, 147). An anti-inflammatory effect targeting the Th17 response might require an abolition of both IL-17 and IL-22 actions. Considering this result and this hypothesis, the inhibition of RORγt, the master driver of Th17 differentiation may be a promising track in asthma.

Overall, substantial progresses are still to be done in the field of treatment targeting the Th17 response in asthma and airway remodeling. Clinical trials relying on relevant outcome criteria and with endotype-based selection of patients are needed. Moreover, timing of administration of biotherapies targeting airway remodeling might be a critical parameter affecting results of clinical trials. Have such therapies to be administered preventively before establishment of severe and fixed (maybe irreversible) airway remodeling and obstruction? Or should we expect to reverse already-established airway remodeling? Further responses are awaited on this burning topic.

## Conclusion

Many evidences are accumulating to indicate a leading role of Th17 cytokines in patients with severe T2^low^ asthma, particularly in patients with neutrophilic asthma and airway remodeling. However, the exact role of Th17 cytokines, especially IL-22, still needs to be clarified *in vivo*, though an important number of experimental *in vitro* data show their involvement in elementary biological processes that contribute to airway remodeling (airway inflammation, subepithelial fibrosis, mucus hyperproduction, thickening of airway smooth muscle, epithelial-mesenchymal transition and alteration of epithelial integrity). Therefore, IL-17 and IL-22 are attractive therapeutic targets for new biotherapies in severe asthma while there is currently no pharmacological treatment targeting airway remodeling. Future clinical trials involving molecules targeting Th17 cytokines using appropriate outcomes and selected populations will be required to progress in this direction.

## Author Contributions

VM-C wrote the first draft of the manuscript. All authors contributed to manuscript revision, read, and approved the submitted version.

## Conflict of Interest

The authors declare that the research was conducted in the absence of any commercial or financial relationships that could be construed as a potential conflict of interest.

## Publisher's Note

All claims expressed in this article are solely those of the authors and do not necessarily represent those of their affiliated organizations, or those of the publisher, the editors and the reviewers. Any product that may be evaluated in this article, or claim that may be made by its manufacturer, is not guaranteed or endorsed by the publisher.

## References

[1] Pearce N Aït-Khaled N Beasley R Mallol J Keil U Mitchell EA. Worldwide trends in the prevalence of asthma symptoms: phase III of the International Study of Asthma and Allergies in Childhood (ISAAC). Thorax. (2007) 62:757–65. 10.1136/thx.2006.070169 17504817 PMC2117323

[2] To T Stanojevic S Moores G Gershon AS Bateman ED Cruz AA. Global asthma prevalence in adults: findings from the cross-sectional world health survey. BMC Public Health. (2012) 12:204. 10.1186/1471-2458-12-204 22429515 PMC3353191

[3] Uphoff EP Bird PK Antó JM Basterrechea M von Berg A Bergström A. Variations in the prevalence of childhood asthma and wheeze in MeDALL cohorts in Europe. ERJ Open Res. (2017) 3:00150–2016. 10.1183/23120541.00150-2016 28845428 PMC5566268

[4] Reddel HK Bateman ED Becker A Boulet LP Cruz AA Drazen JM. A summary of the new GINA strategy: a roadmap to asthma control. Eur Respir J. (2015) 46:622–39. 10.1183/13993003.00853-2015 26206872 PMC4554554

[5] Nordon C Grimaldi-Bensouda L Pribil C Nachbaur G Amzal B Thabut G. Clinical and economic burden of severe asthma: a French cohort study. Respir Med. (2018) 144:42–9. 10.1016/j.rmed.2018.10.002 30366583

[6] Bourdin A Fabry-Vendrand C Ostinelli J Ait-Yahia M Darnal E Bouee S. The burden of severe asthma in france: a case-control study using a medical claims database. J Allergy Clin Immunol Pract. (2019) 7:1477–87. 10.1016/j.jaip.2018.12.029 30685573

[7] Chung KF Wenzel SE Brozek JL Bush A Castro M Sterk PJ. International ERS/ATS guidelines on definition, evaluation and treatment of severe asthma. Eur Respir J. (2014) 43:343–73. 10.1183/09031936.00202013 24337046

[8] Moore WC Meyers DA Wenzel SE Teague WG Li H Li X D'Agostino R. Identification of asthma phenotypes using cluster analysis in the severe asthma research program. Am J Respir Crit Care Med. (2010) 181:315–23. 10.1164/rccm.200906-0896OC 19892860 PMC2822971

[9] Hough KP Curtiss ML Blain TJ Liu RM Trevor J Deshane JS. Airway remodeling in asthma. Front Med. (2020) 7:191. 10.3389/fmed.2020.00191 32509793 PMC7253669

[10] Bergeron C Tulic MK Hamid Q. Airway remodelling in asthma: from benchside to clinical practice. Can Respir J. (2010) 17:318029. 10.1155/2010/318029 20808979 PMC2933777

[11] Bergeron C Al-Ramli W Hamid Q. Remodeling in asthma. Proc Am Thorac Soc. (2009) 6:301–5. 10.1513/pats.200808-089RM 19387034

[12] Girodet PO Ozier A Bara I Tunon De Lara JM Marthan R Berger P. Airway remodeling in asthma: new mechanisms and potential for pharmacological intervention. Pharmacol Ther. (2011) 130:325–37. 10.1016/j.pharmthera.2011.02.001 21334378

[13] Boulet LP Laviolette M Turcotte H Cartier A Dugas M Malo JL. Bronchial subepithelial fibrosis correlates with airway responsiveness to methacholine. Chest. (1997) 112:45–52. 10.1378/chest.112.1.45 9228356

[14] Tepper JS Costa DL Lehmann JR Weber MF Hatch GE. Unattenuated structural and biochemical alterations in the rat lung during functional adaptation to ozone. Am Rev Respir Dis. (1989) 140:493–501. 10.1164/ajrccm/140.2.493 2527482

[15] Minshall E Chakir J Laviolette M Molet S Zhu Z Olivenstein R. IL-11 expression is increased in severe asthma: association with epithelial cells and eosinophils. J Allergy Clin Immunol. (2000) 105:232–8. 10.1016/S0091-6749(00)90070-8 10669841

[16] Benayoun L Druilhe A Dombret MC Aubier M Pretolani M. Airway structural alterations selectively associated with severe asthma. Am J Respir Crit Care Med. (2003) 167:1360–8. 10.1164/rccm.200209-1030OC 12531777

[17] Little SA Sproule MW Cowan MD Macleod KJ Robertson M Love JG. High resolution computed tomographic assessment of airway wall thickness in chronic asthma: reproducibility and relationship with lung function and severity. Thorax. (2002) 57:247–53. 10.1136/thorax.57.3.247 11867830 PMC1746285

[18] Takahashi K Pavlidis S Ng Kee Kwong F Hoda U Rossios C Sun K. Sputum proteomics and airway cell transcripts of current and ex-smokers with severe asthma in U-BIOPRED: an exploratory analysis. Eur Respir J. (2018) 51:2017. 10.1183/13993003.02173-2017 29650557

[19] Agache I Akdis C Jutel M Virchow JC. Untangling asthma phenotypes and endotypes. Allergy Eur J Allergy Clin Immunol. (2012) 67:835–46. 10.1111/j.1398-9995.2012.02832.x 22594878

[20] Shaw DE Sousa AR Fowler SJ Fleming LJ Roberts G Corfield J. Clinical and inflammatory characteristics of the European U-BIOPRED adult severe asthma cohort. Eur Respir J. 46:1308–21. 10.1183/13993003.00779-2015 26357963

[21] Kuruvilla ME Lee FE-H Lee GB. Understanding asthma phenotypes, endotypes, and mechanisms of disease. Clin Rev Allergy Immunol. (2019) 56:219. 10.1007/s12016-018-8712-1 30206782 PMC6411459

[22] Bullone M Carriero V Bertolini F Folino A Mannelli A Stefano AD. Elevated serum IgE, oral corticosteroid dependence and IL-17/22 expression in highly neutrophilic asthma. Eur Respir J. (2019) 54:2019. 10.1183/13993003.00068-2019 31439682

[23] Ray A Kolls JK. Neutrophilic inflammation in asthma and association with disease severity. Trends Immunol. (2017) 38:942–54. 10.1016/j.it.2017.07.003 28784414 PMC5711587

[24] Fajt ML Wenzel SE. Asthma phenotypes and the use of biologic medications in asthma and allergic disease: the next steps toward personalized care. J Allergy Clin Immunol. (2015) 135:299–310. 10.1016/j.jaci.2014.12.1871 25662302

[25] Ricciardolo FLM Sorbello V Folino A Gallo F Massaglia GM Favatà G. Identification of IL-17F/frequent exacerbator endotype in asthma. J Allergy Clin Immunol. (2017) 140:395–406. 10.1016/j.jaci.2016.10.034 27931975

[26] Rupani H Fong WCG Kyyaly MA Kurukulaaratchy RJ. Recent insights into the management of inflammation in asthma. J Inflamm Res. (2021) 14:4371–97. 10.2147/JIR.S295038 34511973 PMC8421249

[27] Lindén A Dahlén B. Interleukin-17 cytokine signalling in patients with asthma. Eur Respir J. (2014) 44:1319–31. 10.1183/09031936.00002314 24925921

[28] Sorbello V Ciprandi G Di Stefano A Massaglia GM Favatà G Conticello S. Nasal IL-17F is related to bronchial IL-17F/neutrophilia and exacerbations in stable atopic severe asthma. Allergy Eur J Allergy Clin Immunol. (2015) 70:236–40. 10.1111/all.12547 25394579

[29] Sze E Bhalla A Nair P. Mechanisms and therapeutic strategies for non-T2 asthma. Allergy Eur J Allergy Clin Immunol. (2020) 75:311–25. 10.1111/all.13985 31309578

[30] Akdis M Palomares O Van De Veen W Van Splunter M Akdis CA. TH17 and TH22 cells: a confusion of antimicrobial response with tissue inflammation versus protection. J Allergy Clin Immunol. (2012) 129:1438–49. 10.1016/j.jaci.2012.05.003 22657405

[31] Cua DJ Sherlock J Chen Y Murphy CA Joyce B Seymour B. Interleukin-23 rather than interleukin-12 is the critical cytokine for autoimmune inflammation of the brain. Nature. (2003) 421:744–8. 10.1038/nature01355 12610626

[32] Harrington LE Mangan PR Weaver CT. Expanding the effector CD4 T-cell repertoire: the Th17 lineage. Curr Opin Immunol. (2006) 18:349–56. 10.1016/j.coi.2006.03.017 16616472

[33] Murphy CA Langrish CL Chen Y Blumenschein W McClanahan T Kastelein RA. Divergent pro- and antiinflammatory roles for IL-23 and IL-12 in joint autoimmune inflammation. J Exp Med. (2003) 198:1951–7. 10.1084/jem.20030896 14662908 PMC2194162

[34] Park H Li Z Yang XO Chang SH Nurieva R Wang YH. A distinct lineage of CD4 T cells regulates tissue inflammation by producing interleukin 17. Nat Immunol. (2005) 6:1133–41. 10.1038/ni1261 16200068 PMC1618871

[35] Bettelli E Korn T Oukka M Kuchroo VK. Induction and effector functions of TH17 cells. Nature. (2008) 453:1051–7. 10.1038/nature07036 18563156 PMC6280661

[36] Duhen T Geiger R Jarrossay D Lanzavecchia A Sallusto F. Production of interleukin 22 but not interleukin 17 by a subset of human skin-homing memory T cells. Nat Immunol. (2009) 10:857–63. 10.1038/ni.1767 19578369

[37] Spits H Artis D Colonna M Diefenbach A Di Santo JP Eberl G. Innate lymphoid cells-a proposal for uniform nomenclature. Nat Rev Immunol. (2013) 13:145–9. 10.1038/nri3365 23348417

[38] Everaere L Ait-Yahia S Molendi-Coste O Vorng H Quemener S LeVu P. Innate lymphoid cells contribute to allergic airway disease exacerbation by obesity. J Allergy Clin Immunol. (2016) 138:1309–18.e11. 10.1016/j.jaci.2016.03.019 27177781

[39] Kim HY Lee HJ Chang YJ Pichavant M Shore SA Fitzgerald KA. Interleukin-17-producing innate lymphoid cells and the NLRP3 inflammasome facilitate obesity-associated airway hyperreactivity. Nat Med. (2014) 20:54–61. 10.1038/nm.3423 24336249 PMC3912313

[40] Molet S Hamid Q Davoine F Nutku E Taha R Pagé N. IL-17 is increased in asthmatic airways and induces human bronchial fibroblasts to produce cytokines. J Allergy Clin Immunol. (2001) 108:430–8. 10.1067/mai.2001.117929 11544464

[41] Hamada H Garcia-Hernandez ML Reome JB Misra SK Strutt TM McKinstry KK. Tc17, a unique subset of CD8 T cells that can protect against lethal influenza challenge. J Immunol. (2009) 182:3469–81. 10.4049/jimmunol.0801814 19265125 PMC2667713

[42] Dudakov JA Hanash AM Van Den Brink MRM. Interleukin-22: immunobiology and pathology. Annu Rev Immunol. (2015) 33:747–85. 10.1146/annurev-immunol-032414-112123 25706098 PMC4407497

[43] Martin B Hirota K Cua DJ Stockinger B Veldhoen M. Interleukin-17-producing γδ T cells selectively expand in response to pathogen products and environmental signals. Immunity. (2009) 31:321–30. 10.1016/j.immuni.2009.06.020 19682928

[44] Sutton CE Lalor SJ Sweeney CM Brereton CF Lavelle EC Mills KHG. Interleukin-1 and IL-23 induce innate IL-17 production from γδ T cells, amplifying Th17 responses and autoimmunity. Immunity. (2009) 31:331–41. 10.1016/j.immuni.2009.08.001 19682929

[45] Simonian PL Wehrmann F Roark CL Born WK O'Brien RL Fontenot AP. γδ T cells protect against lung fibrosis *via* IL-22. J Exp Med. (2010) 207:2239–53. 10.1084/jem.20100061 20855496 PMC2947077

[46] Paget C Ivanov S Fontaine J Renneson J Blanc F Pichavant M. Interleukin-22 is produced by invariant natural killer T lymphocytes during influenza A virus infection: potential role in protection against lung epithelial damages. J Biol Chem. (2012) 287:8816–29. 10.1074/jbc.M111.304758 22294696 PMC3308738

[47] Cosmi L Maggi L Santarlasci V Capone M Cardilicchia E Frosali F. Identification of a novel subset of human circulating memory CD4+ T cells that produce both IL-17A and IL-4. J Allergy Clin Immunol. (2010) 125:12. 10.1016/j.jaci.2009.10.012 20109749

[48] Pennino D Bhavsar PK Effner R Avitabile S Venn P Quaranta M. IL-22 suppresses IFN-γ-mediated lung inflammation in asthmatic patients. J Allergy Clin Immunol. (2013) 131:562–70. 10.1016/j.jaci.2012.09.036 23174657

[49] Zissler UM Chaker AM Effner R Ulrich M Guerth F Piontek G. Interleukin-4 and interferon-γ orchestrate an epithelial polarization in the airways. Mucosal Immunol. (2016) 9:917–26. 10.1038/mi.2015.110 26577568

[50] Ivanov II McKenzie BS Zhou L Tadokoro CE Lepelley A Lafaille JJ. The orphan nuclear receptor RORγt directs the differentiation program of Proinflammatory IL-17+ T helper cells. Cell. (2006) 126:1121–33. 10.1016/j.cell.2006.07.035 16990136

[51] Yang XO Pappu BP Nurieva R Akimzhanov A Kang HS Chung Y. T helper 17 lineage differentiation is programmed by orphan nuclear receptors RORα and RORγ. Immunity. (2008) 28:29–39. 10.1016/j.immuni.2007.11.016 18164222 PMC2587175

[52] Zhou L Ivanov II Spolski R Min R Shenderov K Egawa T. IL-6 programs TH-17 cell differentiation by promoting sequential engagement of the IL-21 and IL-23 pathways. Nat Immunol. (2007) 8:967–74. 10.1038/ni1488 17581537

[53] Yen D Cheung J Scheerens H Poulet F McClanahan T Mckenzie B. IL-23 is essential for T cell-mediated colitis and promotes inflammation *via* IL-17 and IL-6. J Clin Invest. (2006) 116:1310–6. 10.1172/JCI21404 16670770 PMC1451201

[54] Robinson KM Manni ML Biswas PS Alcorn JF. Clinical consequences of targeting IL-17 and TH17 in autoimmune and allergic disorders. Curr Allergy Asthma Rep. (2013) 13:587–95. 10.1007/s11882-013-0361-0 23760974 PMC3812310

[55] Yang XO Panopoulos AD Nurieva R Seon HC Wang D Watowich SS. STAT3 regulates cytokine-mediated generation of inflammatory helper T cells. J Biol Chem. (2007) 282:9358–63. 10.1074/jbc.C600321200 17277312

[56] Korn T Bettelli E Gao W Awasthi A Jäger A Strom TB. IL-21 initiates an alternative pathway to induce proinflammatory T H17 cells. Nature. (2007) 448:484–7. 10.1038/nature05970 17581588 PMC3805028

[57] Dong C. TH17 cells in development: an updated view of their molecular identity and genetic programming. Nat Rev Immunol. (2008) 8:337–48. 10.1038/nri2295 18408735

[58] Gulen MF Kang Z Bulek K Youzhong W Kim TW Chen Y. The receptor SIGIRR suppresses Th17 cell proliferation *via* inhibition of the interleukin-1 receptor pathway and mTOR kinase activation. Immunity. (2010) 32:54–66. 10.1016/j.immuni.2009.12.003 20060329 PMC3015141

[59] Chung Y Chang SH Martinez GJ Yang XO Nurieva R Kang HS. Critical regulation of early Th17 cell differentiation by interleukin-1 signaling. Immunity. (2009) 30:576–87. 10.1016/j.immuni.2009.02.007 19362022 PMC2705871

[60] Chen Q Yang W Gupta S Biswas P Smith P Bhagat G. IRF-4-binding protein inhibits interleukin-17 and interleukin-21 production by controlling the activity of IRF-4 transcription factor. Immunity. (2008) 29:899–911. 10.1016/j.immuni.2008.10.011 19062315 PMC2633410

[61] Manni ML Robinson KM Alcorn JF. A tale of two cytokines: IL-17 and IL-22 in asthma and infection. Expert Rev Respir Med. (2014) 8:25–42. 10.1586/17476348.2014.854167 24325586 PMC4123209

[62] Veldhoen M Hirota K Westendorf AM Buer J Dumoutier L Renauld JC. The aryl hydrocarbon receptor links TH17-cell-mediated autoimmunity to environmental toxins. Nature. (2008) 453:106–9. 10.1038/nature06881 18362914

[63] Stockinger B Hirota K Duarte J Veldhoen M. External influences on the immune system *via* activation of the aryl hydrocarbon receptor. Semin Immunol. (2011) 23:99–105. 10.1016/j.smim.2011.01.008 21288737

[64] Diveu C McGeachy MJ Boniface K Stumhofer JS Sathe M Joyce-Shaikh B. IL-27 blocks RORc expression to inhibit lineage commitment of Th17 cells. J Immunol. (2009) 182:5748–56. 10.4049/jimmunol.0801162 19380822

[65] Ramgolam VS Sha Y Jin J Zhang X Markovic-Plese S. IFN-β inhibits human Th17 cell differentiation. J Immunol. (2009) 183:5418–27. 10.4049/jimmunol.0803227 19783688

[66] Yang XP Ghoreschi K Steward-Tharp SM Rodriguez-Canales J Zhu J Grainger JR. Opposing regulation of the locus encoding IL-17 through direct, reciprocal actions of STAT3 and STAT5. Nat Immunol. (2011) 12:247–54. 10.1038/ni.1995 21278738 PMC3182404

[67] Laurence A Tato CM Davidson TS Kanno Y Chen Z Yao Z. Interleukin-2 signaling *via* STAT5 constrains T helper 17 cell generation. Immunity. (2007) 26:371–81. 10.1016/j.immuni.2007.02.009 17363300

[68] Ohnmacht C Park JH Cording S Wing JB Atarashi K Obata Y. The microbiota regulates type 2 immunity through RORγt+ T cells. Science. (2015) 349:989–93. 10.1126/science.aac4263 26160380

[69] Wang YH Wills-Karp M. The potential role of interleukin-17 in severe asthma. Curr Allergy Asthma Rep. (2011) 11:388–94. 10.1007/s11882-011-0210-y 21773747 PMC4115366

[70] Ramakrishnan RK Al Heialy S Hamid Q. Role of IL-17 in asthma pathogenesis and its implications for the clinic. Expert Rev Respir Med. (2019) 13:1057–68. 10.1080/17476348.2019.1666002 31498708

[71] Eyerich K Eyerich S. Th22 cells in allergic disease. Allergo J Int. (2015) 24:3. 10.1007/s40629-015-0039-3 26120541 PMC4479457

[72] Kolls JK Lindén A. Interleukin-17 family members and inflammation. Immunity. (2004) 21:467–76. 10.1016/j.immuni.2004.08.018 15485625

[73] Laan M Kisand K Kont V Möll K Tserel L Scott HS. Autoimmune regulator deficiency results in decreased expression of CCR4 and CCR7 ligands and in delayed migration of CD4 + thymocytes. J Immunol. (2009) 183:7682–91. 10.4049/jimmunol.0804133 19923453 PMC2795747

[74] Fossiez F Djossou O Chomarat P Flores-Romo L Ait-Yahia S Maat C. T cell interleukin-17 induces stromal cells to produce proinflammatory and hematopoietic cytokines. J Exp Med. (1996) 183:2593–603. 10.1084/jem.183.6.2593 8676080 PMC2192621

[75] Ye P Rodriguez FH Kanaly S Stocking KL Schurr J Schwarzenberger P. Requirement of endogenous stem cell factor and granulocyte-colony-stimulating factor for IL-17-mediated granulopoiesis. J Immunol. (2000) 194:519–27. 10.4049/jimmunol.164.9.4783 10779785

[76] Nembrini C Marsland BJ Kopf M. IL-17-producing T cells in lung immunity and inflammation. J Allergy Clin Immunol. (2009) 123:986–94. 10.1016/j.jaci.2009.03.033 19410688

[77] Pelletier M Maggi L Micheletti A Lazzeri E Tamassia N Costantini C. Evidence for a cross-talk between human neutrophils and Th17 cells. Blood. (2010) 115:335–43. 10.1182/blood-2009-04-216085 19890092

[78] Gaffen SL. Structure and signalling in the IL-17 receptor family. Nat Rev Immunol. (2009) 9:556–67. 10.1038/nri2586 19575028 PMC2821718

[79] Yao Z Fanslow WC Seldin MF Rousseau AM Painter SL Comeau MR. Herpesvirus Saimiri encodes a new cytokine, IL-17, which binds to a novel cytokine receptor. Immunity. (1995) 3:811–21. 10.1016/1074-7613(95)90070-5 8777726

[80] Ho AW Gaffen SL. IL-17RC: a partner in IL-17 signaling and beyond. Semin Immunopathol. (2010) 32:33–42. 10.1007/s00281-009-0185-0 20012905 PMC2837117

[81] Dumoutier L Van Roost E Ameye G Michaux L Renauld J-C. IL-TIF/IL-22: genomic organization and mapping of the human and mouse genes. Genes Immun. (2000) 1:488–94. 10.1038/sj.gene.6363716 11197690

[82] Trifari S Kaplan CD Tran EH Crellin NK Spits H. Identification of a human helper T cell population that has abundant production of interleukin 22 and is distinct from TH-17, TH1 and TH2 cells. Nat Immunol. (2009) 10:864–71. 10.1038/ni.1770 19578368

[83] Lindemans CA Calafiore M Mertelsmann AM O'Connor MH Dudakov JA Jenq RR. Interleukin-22 promotes intestinal-stem-cell-mediated epithelial regeneration. Nature. (2015) 528:560–4. 10.1038/nature16460 26649819 PMC4720437

[84] Hanash AM Dudakov JA Hua G O'Connor MH Young LF Singer N V. Interleukin-22 protects intestinal stem cells from immune-mediated tissue damage and regulates sensitivity to graft versus host disease. Immunity. (2012) 37:339–50. 10.1016/j.immuni.2012.05.028 22921121 PMC3477611

[85] Alcorn JF. IL-22 plays a critical role in maintaining epithelial integrity during pulmonary infection. Front Immunol. (2020) 11:1160. 10.3389/fimmu.2020.01160 32582219 PMC7296169

[86] Boniface K Bernard F-X Garcia M Gurney AL Lecron J-C Morel F. IL-22 inhibits epidermal differentiation and induces proinflammatory gene expression and migration of human keratinocytes. J Immunol. (2005) 174:3695–702. 10.4049/jimmunol.174.6.3695 15749908

[87] Wolk K Sabat R. Interleukin-22: a novel T- and NK-cell derived cytokine that regulates the biology of tissue cells. Cytokine Growth Factor Rev. (2006) 17:367–80. 10.1016/j.cytogfr.2006.09.001 17030002

[88] Wolk K Witte E Wallace E Döcke WD Kunz S Asadullah K. IL-22 regulates the expression of genes responsible for antimicrobial defense, cellular differentiation, and mobility in keratinocytes: a potential role in psoriasis. Eur J Immunol. (2006) 36:1309–23. 10.1002/eji.200535503 16619290

[89] Nograles KE Zaba LC Guttman-Yassky E Fuentes-Duculan J Suárez-Fariñas M Cardinale I. Th17 cytokines interleukin (IL)-17 and IL-22 modulate distinct inflammatory and keratinocyte-response pathways. Br J Dermatol. (2008) 159:1092–102. 10.1111/j.1365-2133.2008.08769.x 18684158 PMC2724264

[90] Wolk K Kunz S Witte E Friedrich M Asadullah K Sabat R. IL-22 increases the innate immunity of tissues. Immunity. (2004) 21:241–54. 10.1016/j.immuni.2004.07.007 15308104

[91] Lee JS Cella M McDonald KG Garlanda C Kennedy GD Nukaya M. AHR drives the development of gut ILC22 cells and postnatal lymphoid tissues *via* pathways dependent on and independent of Notch. Nat Immunol. (2012) 13:144–52. 10.1038/ni.2187 22101730 PMC3468413

[92] Xie MH Aggarwal S Ho WH Foster J Zhang Z Stinson J. Interleukin (IL)-22, a novel human cytokine that signals through the interferon receptor-related proteins CRF2-4 and IL-22R. J Biol Chem. (2000) 275:31335–9. 10.1074/jbc.M005304200 10875937

[93] Kotenko SV Izotova LS Mirochnitchenko OV Esterova E Dickensheets H Donnelly RP. Identification of the functional interleukin-22 (IL-22) receptor complex. The IL-10R2 chain (IL-10Rβ) is a common chain of both the IL-10 and IL-22 (IL-10-related T cell-derived inducible factor, IL-TIF) receptor complexes. J Biol Chem. (2001) 276:2725–32. 10.1074/jbc.M007837200 11035029

[94] Lejeune D Dumoutier L Constantinescu S Kruijer W Schuringa JJ Renauld JC. Interleukin-22 (IL-22) activates the JAK/STAT, ERK, JNK, and p38 MAP kinase pathways in a rat hepatoma cell line: pathways that are shared with and distinct from IL-10. J Biol Chem. (2002) 277:33676–82. 10.1074/jbc.M204204200 12087100

[95] Hirose K Ito T Nakajima H. Roles of IL-22 in allergic airway inflammation in mice and humans. Int Immunol. (2018) 30:413–8. 10.1093/intimm/dxy010 29394345

[96] Aujla SJ Kolls JK. IL-22: a critical mediator in mucosal host defense. J Mol Med. (2009) 87:451–4. 10.1007/s00109-009-0448-1 19219418

[97] Plé C Fan Y Yahia SA Vorng H Everaere L Chenivesse C. Polycyclic aromatic hydrocarbons reciprocally regulate IL-22 and IL-17 cytokines in peripheral blood mononuclear cells from both healthy and asthmatic subjects. PLoS ONE. (2015) 10:e0122372. 10.1371/journal.pone.0122372 25860963 PMC4393221

[98] Sonnenberg GF Nair MG Kirn TJ Zaph C Fouser LA Artis D. Pathological versus protective functions of IL-22 in airway inflammation are regulated by IL-17A. J Exp Med. (2010) 207:1293–305. 10.1084/jem.20092054 20498020 PMC2882840

[99] Besnard AG Sabat R Dumoutier L Renauld JC Willart M Lambrecht B. Dual role of IL-22 in allergic airway inflammation and its cross-talk with IL-17A. Am J Respir Crit Care Med. (2011) 183:1153–63. 10.1164/rccm.201008-1383OC 21297073

[100] Krueger JG Fretzin S Suárez-Fariñas M Haslett PA Phipps KM Cameron GS. IL-17A is essential for cell activation and inflammatory gene circuits in subjects with psoriasis. J Allergy Clin Immunol. (2012) 130:24. 10.1016/j.jaci.2012.04.024 22677045 PMC3470466

[101] Baeten D Baraliakos X Braun J Sieper J Emery P Van Der Heijde D. Anti-interleukin-17A monoclonal antibody secukinumab in treatment of ankylosing spondylitis: a randomised, double-blind, placebo-controlled trial. Lancet. (2013) 382:1705–13. 10.1016/S0140-6736(13)61134-4 24035250

[102] Akiyama S Sakuraba A. Distinct roles of interleukin-17 and T helper 17 cells among autoimmune diseases. J Transl Autoimmun. (2021) 4:100104–100104. 10.1016/j.jtauto.2021.100104 34179741 PMC8188045

[103] Eyerich K Dimartino V Cavani A. IL-17 and IL-22 in immunity: driving protection and pathology. Eur J Immunol. (2017) 47:607–14. 10.1002/eji.201646723 28295238

[104] Wenzel SE Schwartz LB Langmack EL Halliday JL Trudeau JB Gibbs RL. Evidence that severe asthma can be divided pathologically into two inflammatory subtypes with distinct physiologic and clinical characteristics. Am J Respir Crit Care Med. (1999) 160:1001–8. 10.1164/ajrccm.160.3.9812110 10471631

[105] Moore WC Hastie AT Li X Li H Busse WW Jarjour NN Wenzel SE. Sputum neutrophil counts are associated with more severe asthma phenotypes using cluster analysis. J Allergy Clin Immunol. (2014) 133:1557. 10.1016/j.jaci.2013.10.011 24332216 PMC4040309

[106] Al-Ramli W Préfontaine D Chouiali F Martin JG Olivenstein R Lemière C. TH17-associated cytokines (IL-17A and IL-17F) in severe asthma. J Allergy Clin Immunol. (2009) 123:1185–7. 10.1016/j.jaci.2009.02.024 19361847

[107] Doe C Bafadhel M Siddiqui S Desai D Mistry V Rugman P. Expression of the T helper 17-associated cytokines IL-17A and IL-17F in asthma and COPD. Chest. (2010) 138:1140–7. 10.1378/chest.09-3058 20538817 PMC2972626

[108] Lamb D De Sousa D Quast K Fundel-Clemens K Erjefält JS Sandén C. RORγt inhibitors block both IL-17 and IL-22 conferring a potential advantage over anti-IL-17 alone to treat severe asthma. Respir Res. (2021) 22:7. 10.1186/s12931-021-01743-7 34022896 PMC8141258

[109] Agache I Ciobanu C Agache C Anghel M. Increased serum IL-17 is an independent risk factor for severe asthma. Respir Med. (2010) 104:1131–7. 10.1016/j.rmed.2010.02.018 20338742

[110] Bullens DMA Truyen E Coteur L Dilissen E Hellings PW Dupont LJ. IL-17 mRNA in sputum of asthmatic patients: linking T cell driven inflammation and granulocytic influx? Respir Res. (2006) 7:135. 10.1186/1465-9921-7-135 17083726 PMC1636037

[111] Irvin C Zafar I Good J Rollins D Christianson C Gorska MM. Increased frequency of dual-positive TH2/TH17 cells in bronchoalveolar lavage fluid characterizes a population of patients with severe asthma. J Allergy Clin Immunol. (2014) 134:1175–86.e7. 10.1016/j.jaci.2014.05.038 25042748 PMC4254017

[112] Wilson RH Whitehead GS Nakano H Free ME Kolls JK Cook DN. Allergic sensitization through the airway primes Th17-dependent neutrophilia and airway hyperresponsiveness. Am J Respir Crit Care Med. (2009) 180:720. 10.1164/rccm.200904-0573OC 19661246 PMC2778149

[113] He R Oyoshi MK Jin H Geha RS. Epicutaneous antigen exposure induces a Th17 response that drives airway inflammation after inhalation challenge. Proc Natl Acad Sci USA. (2007) 104:15817–22. 10.1073/pnas.0706942104 17893340 PMC2000444

[114] Ano S Morishima Y Ishii Y Yoh K Yageta Y Ohtsuka S. Transcription factors GATA-3 and RORγt are important for determining the phenotype of allergic airway inflammation in a murine model of asthma. J Immunol. (2013) 190:1056–65. 10.4049/jimmunol.1202386 23293351

[115] McKinley L Alcorn JF Peterson A DuPont RB Kapadia S Logar A. TH17 cells mediate steroid-resistant airway inflammation and airway hyperresponsiveness in mice. J Immunol. (2008) 181:4089–97. 10.4049/jimmunol.181.6.4089 18768865 PMC3638757

[116] Wu Y Yue J Wu J Zhou W Li D Ding K. Obesity may provide Pro-ILC3 development inflammatory environment in asthmatic children. J Immunol Res. (2018) 2018:1628620. 10.1155/2018/1628620 30622974 PMC6304845

[117] Hellings PW Kasran A Liu Z Vandekerckhove P Wuyts A Overbergh L. Interleukin-17 orchestrates the granulocyte influx into airways after allergen inhalation in a mouse model of allergic asthma. Am J Respir Cell Mol Biol. (2003) 28:42–50. 10.1165/rcmb.4832 12495931

[118] Wang Q Li H Yao Y Xia D Zhou J. The overexpression of heparin-binding epidermal growth factor is responsible for Th17-induced airway remodeling in an experimental asthma model. J Immunol. (2010) 185:834–41. 10.4049/jimmunol.0901490 20530256

[119] Zhao J Lloyd CM Noble A. Th17 responses in chronic allergic airway inflammation abrogate regulatory T-cell-mediated tolerance and contribute to airway remodeling. Mucosal Immunol. (2013) 6:335–46. 10.1038/mi.2012.76 22892938 PMC4233308

[120] Camargo LN Righetti RF Aristóteles LR dos Santos TM de Souza FCR Fukuzaki S. Effects of anti-IL-17 on inflammation, remodeling, and oxidative stress in an experimental model of asthma exacerbated by LPS. Front Immunol. (2018) 8:1835. 10.3389/fimmu.2017.01835 29379497 PMC5760512

[121] Johnson JR Nishioka M Chakir J Risse PA Almaghlouth I Bazarbashi AN. IL-22 contributes to TGF-β1-mediated epithelial-mesenchymal transition in asthmatic bronchial epithelial cells. Respir Res. (2013) 14:1–12. 10.1186/1465-9921-14-118 24283210 PMC4176096

[122] Tamasauskiene L Gintauskiene VM Bastyte D Sitkauskiene B. Role of IL-22 in persistent allergic airway diseases caused by house dust mite: a pilot study. BMC Pulm Med. (2021) 21:1–8. 10.1186/s12890-021-01410-z 33478443 PMC7819229

[123] Liu J Shang B Bai J. IL-22/IL-22R1 promotes proliferation and collagen synthesis of MRC-5 cells *via* the JAK/STAT3 signaling pathway and regulates airway subepithelial fibrosis. Exp Ther Med. (2020) 20:2148–56. 10.3892/etm.2020.8931 32765690 PMC7401847

[124] Farfariello V Amantini C Nabissi M Morelli MB Aperio C Caprodossi S. IL-22 mRNA in peripheral blood mononuclear cells from allergic rhinitic and asthmatic pediatric patients. Pediatr Allergy Immunol. (2011) 22:419–23. 10.1111/j.1399-3038.2010.01116.x 21535180

[125] Ito T Hirose K Nakajima H. Bidirectional roles of IL-22 in the pathogenesis of allergic airway inflammation. Allergol Int. (2019) 68:4–8. 10.1016/j.alit.2018.10.002 30424940

[126] Arshad T Mansur F Palek R Manzoor S Liska V. A double edged sword role of interleukin-22 in wound healing and tissue regeneration. Front Immunol. (2020) 11:2148. 10.3389/fimmu.2020.02148 33042126 PMC7527413

[127] Takahashi K Hirose K Kawashima S Niwa Y Wakashin H Iwata A. IL-22 attenuates IL-25 production by lung epithelial cells and inhibits antigen-induced eosinophilic airway inflammation. J Allergy Clin Immunol. (2011) 128:18. 10.1016/j.jaci.2011.06.018 21794904

[128] Taube C Tertilt C Gyülveszi G Dehzad N Kreymborg K Schneeweiss K. IL-22 is produced by innate lymphoid cells and limits inflammation in allergic airway disease. PLoS ONE. (2011) 6:e21799. 10.1371/journal.pone.0021799 21789181 PMC3138740

[129] Nakagome K Imamura M Kawahata K Harada H Okunishi K Matsumoto T. High expression of IL-22 suppresses antigen-induced immune responses and eosinophilic airway inflammation *via* an IL-10–associated mechanism. J Immunol. (2011) 187:5077–89. 10.4049/jimmunol.1001560 21998459

[130] Fang P Zhou L Zhou Y Kolls JK Zheng T Zhu Z. Immune modulatory effects of IL-22 on allergen-induced pulmonary inflammation. PLoS ONE. (2014) 9:e107454. 10.1371/journal.pone.0107454 25254361 PMC4177833

[131] Ito T Hirose K Saku A Kono K Takatori H Tamachi T. IL-22 induces Reg3γ and inhibits allergic inflammation in house dust mite-induced asthma models. J Exp Med. (2017) 214:3037–50. 10.1084/jem.20162108 28811323 PMC5626396

[132] Leyva-Castillo JM Yoon J Geha RS. IL-22 promotes allergic airway inflammation in epicutaneously sensitized mice. J Allergy Clin Immunol. (2019) 143:619–30.e7. 10.1016/j.jaci.2018.05.032 29920352 PMC6298864

[133] Lou H Lu J Choi EB Oh MH Jeong M Barmettler S. Expression of IL-22 in the skin causes Th2-biased immunity, epidermal barrier dysfunction, and pruritus *via* stimulating epithelial Th2 cytokines and the GRP pathway. J Immunol. (2017) 198:2543–55. 10.4049/jimmunol.1600126 28228560 PMC5360537

[134] Glocova I Brück J Geisel J Müller-Hermelink E Widmaier K Yazdi AS. Induction of skin-pathogenic Th22 cells by epicutaneous allergen exposure. J Dermatol Sci. (2017) 87:268–77. 10.1016/j.jdermsci.2017.06.006 28655472

[135] Nanzer AM Chambers ES Ryanna K Richards DF Black C Timms PM. Enhanced production of IL-17A in patients with severe asthma is inhibited by 1α,25-dihydroxyvitamin D3 in a glucocorticoid-independent fashion. J Allergy Clin Immunol. (2013) 132:37. 10.1016/j.jaci.2013.03.037 23683514

[136] Zhu J Cao Y Li K Wang Z Zuo P Xiong W. Increased expression of aryl hydrocarbon receptor and interleukin 22 in patients with allergic asthma. Asian Pacific J Allergy Immunol. (2011) 29:266–72. 22053597

[137] Bouté M Ait Yahia S Nanou J Alvarez-Simon D Audousset C Vorng H. Direct activation of the aryl hydrocarbon receptor by dog allergen participates in airway neutrophilic inflammation. Allergy Eur J Allergy Clin Immunol. (2021) 76:2245–9. 10.1111/all.14740 33465835

[138] Badi YE Pavel AB Pavlidis S Riley JH Bates S Kermani NZ. Mapping atopic dermatitis and anti–IL-22 response signatures to type 2–low severe neutrophilic asthma. J Allergy Clin Immunol. (2021) 149:89–101. 10.1016/j.jaci.2021.04.010 33891981

[139] Ciprandi G Cuppari C Salpietro AM Tosca MA Rigoli L Grasso L. Serum IL-23 strongly and inversely correlates with FEV1 in asthmatic children. Int Arch Allergy Immunol. (2012) 159:183–6. 10.1159/000336418 22678234

[140] Louis R Lau LCK Bron AO Roldaan AC Radermecker M Djukanović R. The relationship between airways inflammation and asthma severity. Am J Respir Crit Care Med. (2000) 161:9–16. 10.1164/ajrccm.161.1.9802048 10619791

[141] Cundall M Sun Y Miranda C Trudeau JB Barnes S Wenzel SE. Neutrophil-derived matrix metalloproteinase-9 is increased in severe asthma and poorly inhibited by glucocorticoids. J Allergy Clin Immunol. (2003) 112:1064–71. 10.1016/j.jaci.2003.08.013 14657859

[142] Jatakanon A Uasuf C Maziak W Lim S Chung KF Barnes PJ. Neutrophilic inflammation in severe persistent asthma. Am J Respir Crit Care Med. (1999) 160:1532–9. 10.1164/ajrccm.160.5.9806170 10556116

[143] Dragon S Rahman MS Yang J Unruh H Halayko AJ Gounni AS. IL-17 enhances IL-1β-mediated CXCL-8 release from human airway smooth muscle cells. Am J Physiol Lung Cell Mol Physiol. (2007) 292:2006. 10.1152/ajplung.00306.2006 17189320

[144] Wolk K Haugen HS Xu W Witte E Waggie K Anderson M. IL-22 and IL-20 are key mediators of the epidermal alterations in psoriasis while IL-17 and IFN-γ are not. J Mol Med. (2009) 87:523–36. 10.1007/s00109-009-0457-0 19330474

[145] Stacey MA Marsden M Pham N TA Clare S Dolton G Stack G. Neutrophils recruited by IL-22 in peripheral tissues function as TRAIL-dependent antiviral effectors against MCMV. Cell Host Microbe. (2014) 15:471–83. 10.1016/j.chom.2014.03.003 24721575 PMC3989063

[146] Lilly LM Gessner MA Dunaway CW Metz AE Schwiebert L Weaver CT. The β-glucan receptor dectin-1 promotes lung immunopathology during fungal allergy *via* IL-22. J Immunol. (2012) 189:3653–60. 10.4049/jimmunol.1201797 22933634 PMC3448838

[147] Yang L Zheng Y Miao Y Yan W Geng Y Dai Y. Bergenin, a PPARγ agonist, inhibits Th17 differentiation and subsequent neutrophilic asthma by preventing GLS1-dependent glutaminolysis. Acta Pharmacol Sin. (2021) 21:717. 10.1038/s41401-021-00717-1 34267342 PMC8975945

[148] Ventura I Vega A Chacõn P Chamorro C Aroca R Gõmez E. Neutrophils from allergic asthmatic patients produce and release metalloproteinase-9 upon direct exposure to allergens. Allergy Eur J Allergy Clin Immunol. (2014) 69:898–905. 10.1111/all.12414 24773508

[149] Voynow JA Shinbashi M. Neutrophil elastase and chronic lung disease. Biomolecules. (2021) 11:65. 10.3390/biom11081065 34439732 PMC8394930

[150] Michaudel C Bataille F Maillet I Fauconnier L Colas C Sokol H Straube M. Ozone-induced aryl hydrocarbon receptor activation controls lung inflammation *via* interleukin-22 modulation. Front Immunol. (2020) 11:144. 10.3389/fimmu.2020.00144 32161582 PMC7053361

[151] Zheng Y Danilenko DM Valdez P Kasman I Eastham-Anderson J Wu J. Interleukin-22, a TH17 cytokine, mediates IL-23-induced dermal inflammation and acanthosis. Nature. (2007) 445:648–51. 10.1038/nature05505 17187052

[152] Starkey MR Plank MW Casolari P Papi A Pavlidis S Guo Y. IL-22 and its receptors are increased in human and experimental COPD and contribute to pathogenesis. Eur Respir J. (2019) 54:2018. 10.1183/13993003.00174-2018 31196943 PMC8132110

[153] Yu Y Zhao L Xie Y Xu Y Jiao W Wu J. Th1/th17 cytokine profiles are associated with disease severity and exacerbation frequency in copd patients. Int J COPD. (2020) 15:1287–99. 10.2147/COPD.S252097 32606639 PMC7294048

[154] Papp KA Leonardi C Menter A Ortonne J-P Krueger JG Kricorian G. Brodalumab, an anti–interleukin-17–receptor antibody for psoriasis. N Engl J Med. (2012) 366:1181–9. 10.1056/NEJMoa1109017 22455412

[155] Simon D Aeberhard C Erdemoglu Y Simon HU. Th17 cells and tissue remodeling in atopic and contact dermatitis. Allergy Eur J Allergy Clin Immunol. (2014) 69:125–31. 10.1111/all.12351 24372156

[156] Eyerich S Eyerich K Pennino D Carbone T Nasorri F Pallotta S. Th22 cells represent a distinct human T cell subset involved in epidermal immunity and remodeling. J Clin Invest. (2009) 119:3573–85. 10.1172/JCI40202 19920355 PMC2786807

[157] Ji X Li J Xu L Wang W Luo M Luo S. IL4 and IL-17A provide a Th2/Th17-polarized inflammatory milieu in favor of TGF-β1 to induce bronchial epithelial-mesenchymal transition (EMT). Int J Clin Exp Pathol. (2013) 6:1481–92. Available online at: /pmc/articles/PMC3726963/ (accessed January 5, 2022). 23923066 PMC3726963

[158] Brunner PM Pavel AB Khattri S Leonard A Malik K Rose S. Baseline IL-22 expression in patients with atopic dermatitis stratifies tissue responses to fezakinumab. J Allergy Clin Immunol. (2019) 143:142–54. 10.1016/j.jaci.2018.07.028 30121291

[159] Habener A Happle C Grychtol R Skuljec J Busse M Dalüge K. Regulatory B cells control airway hyperreactivity and lung remodeling in a murine asthma model. J Allergy Clin Immunol. (2021) 147:2281–94.e7. 10.1016/j.jaci.2020.09.041 33249168

[160] Patakas A Benson RA Withers DR Conigliaro P McInnes IB Brewer JM. Th17 effector cells support B cell responses outside of germinal centres. PLoS ONE. (2012) 7:49715. 10.1371/journal.pone.0049715 23166752 PMC3500323

[161] Mitsdoerffer M Lee Y Jäger A Kim HJ Korn T Kolls JK. Proinflammatory T helper type 17 cells are effective B-cell helpers. Proc Natl Acad Sci USA. (2010) 107:14292–7. 10.1073/pnas.1009234107 20660725 PMC2922571

[162] Halwani R Al-Kufaidy R Vazquez-Tello A Pureza MA BaHammam AS Al-Jahdali H. IL-17 enhances chemotaxis of primary human B cells during asthma. PLoS ONE. (2014) 9:114604. 10.1371/journal.pone.0114604 25494178 PMC4262428

[163] Scanlon KM Hawksworth RJ Lane SJ Mahon BP. IL-17A induces CCL28, supporting the chemotaxis of IgE-secreting B cells. Int Arch Allergy Immunol. (2011) 156:51–61. 10.1159/000322178 21447959

[164] Al-Kufaidy R Vazquez-Tello A BaHammam AS Al-Muhsen S Hamid Q Halwani R. IL-17 enhances the migration of B cells during asthma by inducing CXCL13 chemokine production in structural lung cells. J Allergy Clin Immunol. (2017) 139:696–9.e5. 10.1016/j.jaci.2016.07.037 27639935

[165] Barone F Nayara S Camposa J Cloakea T Withers DR Toellner KM. IL-22 regulates lymphoid chemokine production and assembly of tertiary lymphoid organs. Proc Natl Acad Sci USA. (2015) 112:11024–9. 10.1073/pnas.1503315112 26286991 PMC4568258

[166] Rheum A Corneth O Mus A-M Asmawidjaja P Ouyang W Kil L. Impaired B cell immunity in IL-22 knock-out mice in collagen induced arthritis. Ann Rheum Dis. (2011) 70:A58–9. 10.1136/ard.2010.149005.328850992

[167] Kuo C Lim S King NJC Bartlett NW Walton RP Zhu J. Rhinovirus infection induces expression of airway remodelling factors *in vitro* and *in vivo*. Respirology. (2011) 16:367–77. 10.1111/j.1440-1843.2010.01918.x 21199160

[168] Becnel D You D Erskin J Dimina DM Cormier SA. A role for airway remodeling during respiratory syncytial virus infection. Respir Res. (2005) 6:1–11. 10.1186/1465-9921-6-122 16242038 PMC1283984

[169] Lloyd CM Robinson DS. Allergen-induced airway remodelling. Eur Respir J. (2007) 29:1020–32. 10.1183/09031936.00150305 17470623 PMC3384680

[170] Rahmawati SF te Velde M Kerstjens HAM Dömling ASS Groves MR Gosens R. Pharmacological rationale for targeting IL-17 in asthma. Front Allergy. (2021) 2:40. 10.3389/falgy.2021.694514 35387016 PMC8974835

[171] Nightingale S. Role of IL-17 and IL-23 in the pathogenesis of neutrophilic asthma. Int J Immunol Immunother. (2020) 7:1410049. 10.23937/2378-3672/141004929079726

[172] Hirose K Takahashi K Nakajima H. Roles of IL-22 in allergic airway inflammation. J Allergy. (2013) 2013:1–5. 10.1155/2013/260518 23577040 PMC3594983

[173] Filidou E Valatas V Drygiannakis I Arvanitidis K Vradelis S Kouklakis G. Cytokine receptor profiling in human colonic subepithelial myofibroblasts: a differential effect of Th polarization-associated cytokines in intestinal fibrosis. Inflamm Bowel Dis. (2018) 24:2224–41. 10.1093/ibd/izy204 29860326

[174] Komai M Tanaka H Masuda T Nagao K Ishizaki M Sawada M. Role of Th2 responses in the development of allergen-induced airway remodelling in a murine model of allergic asthma. Br J Pharmacol. (2003) 138:912–20. 10.1038/sj.bjp.0705105 12642393 PMC1573716

[175] Bellini A Marini MA Bianchetti L Barczyk M Schmidt M Mattoli S. Interleukin (IL)-4, IL-13, and IL-17A differentially affect the profibrotic and proinflammatory functions of fibrocytes from asthmatic patients. Mucosal Immunol. (2012) 5:140–9. 10.1038/mi.2011.60 22189956

[176] Hayashi H Kawakita A Okazaki S Yasutomi M Murai H Ohshima Y. IL-17A/F modulates fibrocyte functions in cooperation with CD40-mediated signaling. Inflammation. (2013) 36:830–8. 10.1007/s10753-013-9609-z 23400328

[177] Al-Muhsen S Letuve S Vazquez-Tello A Pureza MA Al-Jahdali H Bahammam AS Hamid Q. Th17 cytokines induce pro-fibrotic cytokines release from human eosinophils. Respir Res. (2013) 14:34. 10.1186/1465-9921-14-34 23496774 PMC3602055

[178] Ramakrishnan RK Bajbouj K Al Heialy S Mahboub B Ansari AW Hachim IY. IL-17 induced autophagy regulates mitochondrial dysfunction and fibrosis in severe asthmatic bronchial fibroblasts. Front Immunol. (2020) 11:1002. 10.3389/fimmu.2020.01002 32670268 PMC7326148

[179] Loubaki L Hadj-Salem I Fakhfakh R Jacques E Plante S Boisvert M. Co-culture of human bronchial fibroblasts and CD4+ T cells increases Th17 cytokine signature. PLoS ONE. (2013) 8:e81983. 10.1371/journal.pone.0081983 24349168 PMC3857794

[180] Hoshino H Laan M Sjöstrand M Lötvall J Skoogh BE Lindén A. Increased elastase and myeloperoxidase activity associated with neutrophil recruitment by IL-17 in airways *in vivo*. J Allergy Clin Immunol. (2000) 105:143–9. 10.1016/S0091-6749(00)90189-1 10629464

[181] Chakir J Shannon J Molet S Fukakusa M Elias J Laviolette M. Airway remodeling-associated mediators in moderate to severe asthma: effect of steroids on TGF-β, IL-11, IL-17, and type I and type III collagen expression. J Allergy Clin Immunol. (2003) 111:1293–8. 10.1067/mai.2003.1557 12789232

[182] Chen Y Thai P Zhao YH Ho YS DeSouza MM Wu R. Stimulation of airway mucin gene expression by interleukin (IL)-17 through IL-6 paracrine/autocrine loop. J Biol Chem. (2003) 278:17036–43. 10.1074/jbc.M210429200 12624114

[183] Fujisawa T Velichko S Thai P Hung L-Y Huang F Wu R. Regulation of airway MUC5AC expression by IL-1β and IL-17A; the NF-κB paradigm. J Immunol. (2009) 183:6236–43. 10.4049/jimmunol.0900614 19841186 PMC4623590

[184] Mammen MJ Ali J Aurora A Sharma UC Aalinkeel R Mahajan SD et L. IL-17 is a key regulator of mucin-galectin-3 interactions in asthma. Int J Cell Biol. (2021) 2021:9997625. 10.1155/2021/9997625 34221020 PMC8211528

[185] Herbert C Shadie AM Kumar RK. Interleukin-17 signalling in a murine model of mild chronic asthma. Int Arch Allergy Immunol. (2013) 162:253–62. 10.1159/000353247 24022125

[186] Newcomb DC Boswell MG Sherrill TP Polosukhin VV Boyd KL Goleniewska K. IL-17A induces signal transducers and activators of transcription-6- independent airway mucous cell metaplasia. Am J Respir Cell Mol Biol. (2013) 48:711–6. 10.1165/rcmb.2013-0017OC 23392574 PMC3727878

[187] Pezzulo AA Tudas RA Stewart CG Vargas Buonfiglio LG Lindsay BD Taft PJ. HSP90 inhibitor geldanamycin reverts IL-13–and IL-17–induced airway goblet cell metaplasia. J Clin Invest. (2019) 129:744–58. 10.1172/JCI123524 30640172 PMC6355221

[188] Sohal SS Ward C Walters EH. Importance of epithelial mesenchymal transition (EMT) in COPD and asthma. Thorax. (2014) 69:768. 10.1136/thoraxjnl-2014-205582 24842787

[189] Wang T Liu Y Zou JF Cheng ZS. Interleukin-17 induces human alveolar epithelial to mesenchymal cell transition *via* the TGF-β1 mediated Smad2/3 and ERK1/2 activation. PLoS ONE. (2017) 12:183972. 10.1371/journal.pone.0183972 28873461 PMC5584923

[190] Gu K Li M-M Shen J Liu F Cao J-Y Jin S. Interleukin-17-induced EMT promotes lung cancer cell migration and invasion *via* NF-κB/ZEB1 signal pathway. Am J Cancer Res. (2015) 5:1169–79. 26045995 PMC4449444

[191] Ma L Jiang M Zhao X Sun J Pan Q Chu S. Cigarette and IL-17A synergistically induce bronchial epithelial-mesenchymal transition *via* activating IL-17R/NF-κB signaling. BMC Pulm Med. (2020) 20:1–7. 10.1186/s12890-020-1057-6 32000730 PMC6993491

[192] Jiang G Liu CT Zhang WD. IL-17A and GDF15 are able to induce epithelial-mesenchymal transition of lung epithelial cells in response to cigarette smoke. Exp Ther Med. (2018) 16:12–20. 10.3892/etm.2018.6145 29977354 PMC6030931

[193] Vittal R Fan L Greenspan DS Mickler EA Gopalakrishnan B Gu H. IL-17 induces type V collagen overexpression and EMT *via* TGF-β-dependent pathways in obliterative bronchiolitis. Am J Physiol Lung Cell Mol Physiol. (2013) 304:L401. 10.1152/ajplung.00080.2012 23262228 PMC3602743

[194] Sun J Chu S Lu M Pan Q Li D Zheng S. The roles of dipeptidyl peptidase-4 and its inhibitors in the regulation of airway epithelial-mesenchymal transition. Exp Lung Res. (2020) 46:163–73. 10.1080/01902148.2020.1753853 32292085

[195] Haddad A Gaudet M Plesa M Allakhverdi Z Mogas AK Audusseau S. Neutrophils from severe asthmatic patients induce epithelial to mesenchymal transition in healthy bronchial epithelial cells. Respir Res. (2019) 20:1–14. 10.1186/s12931-019-1186-8 31665016 PMC6819645

[196] Chang Y Al-Alwan L Risse PA Halayko AJ Martin JG Baglole CJ. Th17-associated cytokines promote human airway smooth muscle cell proliferation. FASEB J. (2012) 26:5152–60. 10.1096/fj.12-208033 22898922

[197] Chang Y Al-Alwan L Risse PA Roussel L Rousseau S Halayko AJ. TH17 cytokines induce human airway smooth muscle cell migration. J Allergy Clin Immunol. (2011) 127:1046–53.e2. 10.1016/j.jaci.2010.12.1117 21345484

[198] Al-Alwan LA Chang Y Baglole CJ Risse PA Halayko AJ Martin JG. Autocrine-regulated airway smooth muscle cell migration is dependent on IL-17-induced growth-related oncogenes. J Allergy Clin Immunol. (2012) 130:977–85.e6. 10.1016/j.jaci.2012.04.042 22698519

[199] Ogawa H Azuma M Tsunematsu T Morimoto Y Kondo M Tezuka T. Neutrophils induce smooth muscle hyperplasia *via* neutrophil elastase-induced FGF-2 in a mouse model of asthma with mixed inflammation. Clin Exp Allergy. (2018) 48:1715–25. 10.1111/cea.13263 30171733

[200] Kudo M Melton AC Chen C Engler MB Huang KE Ren X. IL-17A produced by αβ T cells drives airway hyper-responsiveness in mice and enhances mouse and human airway smooth muscle contraction. Nat Med. (2012) 18:547–54. 10.1038/nm.2684 22388091 PMC3321096

[201] Chiba Y Tanoue G Suto R Suto W Hanazaki M Katayama H. Interleukin-17A directly acts on bronchial smooth muscle cells and augments the contractility. Pharmacol Rep. (2017) 69:377–85. 10.1016/j.pharep.2016.12.007 28267638

[202] Fong V Hsu A Wu E Looney AP Ganesan P Ren X. Arhgef12 drives IL17A-induced airway contractility and airway hyperresponsiveness in mice. JCI Insight. (2018) 3:123578. 10.1172/jci.insight.123578 30385725 PMC6238747

[203] Jendzjowsky N Laing A Malig M Matyas J Heuvel E Dumonceaux C. Long-term modulation of airway remodeling in severe asthma following bronchial thermoplasty. Eur Respir J. (2021) 2021:2100622. 10.1183/13993003.00622-2021 34049950

[204] Menzies-Gow A Corren J Bourdin A Chupp G Israel E Wechsler ME. Tezepelumab in adults and adolescents with severe, uncontrolled asthma. N Engl J Med. (2021) 384:1800–9. 10.1056/NEJMoa2034975 33979488

[205] Diver S Khalfaoui L Emson C Wenzel SE Menzies-Gow A Wechsler ME. Effect of tezepelumab on airway inflammatory cells, remodelling, and hyperresponsiveness in patients with moderate-to-severe uncontrolled asthma (CASCADE): a double-blind, randomised, placebo-controlled, phase 2 trial. Lancet Respir Med. (2021) 9:1299–312. 10.1016/S2213-2600(21)00226-5 34256031

[206] Busse WW Holgate S Kerwin E Chon Y Feng JY Lin J. Randomized, double-blind, placebo-controlled study of brodalumab, a human anti-IL-17 receptor monoclonal antibody, in moderate to severe asthma. Am J Respir Crit Care Med. (2013) 188:1294–302. 10.1164/rccm.201212-2318OC 24200404

[207] Staton TL Peng K Owen R Choy DF Cabanski CR Fong A. A phase I, randomized, observer-blinded, single and multiple ascending-dose study to investigate the safety, pharmacokinetics, and immunogenicity of BITS7201A, a bispecific antibody targeting IL-13 and IL-17, in healthy volunteers. BMC Pulm Med. (2019) 19:9. 10.1186/s12890-018-0763-9 30616547 PMC6323662

[208] Brightling CE Nair P Cousins DJ Louis R Singh D. Risankizumab in severe asthma — a phase 2a, placebo-controlled trial. N Engl J Med. (2021) 385:1669–79. 10.1056/NEJMoa2030880 34706172

[209] Hynes GM Hinks TSC. The role of interleukin-17 in asthma: a protective response? ERJ Open Res. (2020) 6:00364–2019. 10.1183/23120541.00364-2019 32494573 PMC7248344

[210] Chesné J Braza F Mahay G Brouard S Aronica M Magnan A. IL-17 in severe asthma: where do we stand? Am J Respir Crit Care Med. (2014) 190:1094–101. 10.1164/rccm.201405-0859PP 25162311

[211] Ouyang S Liu C Xiao J Chen X Lui AC Li X. Targeting IL-17A/glucocorticoid synergy to CSF3 expression in neutrophilic airway diseases. JCI Insight. (2020) 5:132836. 10.1172/jci.insight.132836 32051346 PMC7098787

[212] Ciprandi G Fenoglio D De Amici M Marseglia G Murdaca G Di Gioacchino M. Serum IL-17 after one course of sublingual immunotherapy in allergic rhinitis to birch. Eur J Inflamm. (2009) 7:49–51. 10.1177/1721727X0900700107

[213] Li CW Lu HG Chen DH Lin Z Bin A Wang DY. *In vivo* and *in vitro* studies of Th17 response to specific immunotherapy in house dust mite-induced allergic rhinitis patients. PLoS ONE. (2014) 9:e91950. 10.1371/journal.pone.0091950 24647473 PMC3960160

[214] Zissler UM Jakwerth CA Guerth FM Pechtold L Aguilar-Pimentel JA Dietz K. Early IL-10 producing B-cells and coinciding Th/Tr17 shifts during three year grass-pollen AIT. EBioMedicine. (2018) 36:475–88. 10.1016/j.ebiom.2018.09.016 30318182 PMC6197437

[215] Zissler UM Schmidt-Weber CB. Predicting success of allergen-specific immunotherapy. Front Immunol. (2020) 11:1826. 10.3389/fimmu.2020.01826 32983092 PMC7477353

[216] Wang J Qiu L Chen Y Chen M. Sublingual immunotherapy increases Treg/Th17 ratio in allergic rhinitis. Open Med. (2021) 16:826–32. 10.1515/med-2021-0285 34056115 PMC8142385

[217] Wang SH Zissler UM Buettner M Heine S Heldner A Kotz S. An exhausted phenotype of TH2 cells is primed by allergen exposure, but not reinforced by allergen-specific immunotherapy. Allergy Eur J Allergy Clin Immunol. (2021) 76:2827–39. 10.1111/all.14896 33969495

[218] Mcalees JW Lajoie S Dienger K Sproles AA Richgels PK Yang Y. Differential control of CD4+ T-cell subsets by the PD-1/PD-L1 axis in a mouse model of allergic asthma. Eur J Immunol. (2015) 45:1019–29. 10.1002/eji.201444778 25630305 PMC4440042

[219] Akbari O Stock P Singh AK Lombardi V Lee WL Freeman GJ. PD-L1 and PD-L2 modulate airway inflammation and iNKT-cell-dependent airway hyperreactivity in opposing directions. Mucosal Immunol. (2010) 3:81–91. 10.1038/mi.2009.112 19741598 PMC2845714

[220] Miyamoto C Kojo S Yamashita M Moro K Lacaud G Shiroguchi K. Runx/Cbfβ complexes protect group 2 innate lymphoid cells from exhausted-like hyporesponsiveness during allergic airway inflammation. Nat Commun. (2019) 10:1–13. 10.1038/s41467-019-08365-0 30683858 PMC6347616

[221] Ebihara T Taniuchi I. Exhausted-like group 2 innate lymphoid cells in chronic allergic inflammation. Trends Immunol. (2019) 40:1095–104. 10.1016/j.it.2019.10.007 31735510

[222] Guttman-Yassky E Brunner PM Neumann AU Khattri S Pavel AB Malik K. Efficacy and safety of fezakinumab (an IL-22 monoclonal antibody) in adults with moderate-to-severe atopic dermatitis inadequately controlled by conventional treatments: a randomized, double-blind, phase 2a trial. J Am Acad Dermatol. (2018) 78:872–81.e6. 10.1016/j.jaad.2018.01.016 29353025 PMC8711034

